# Fatty acid profile of Romanian’s common bean (*Phaseolus vulgaris* L.) lipid fractions and their complexation ability by β-cyclodextrin

**DOI:** 10.1371/journal.pone.0225474

**Published:** 2019-11-22

**Authors:** Ioan David, Manuela D. Orboi, Marius D. Simandi, Cosmina A. Chirilă, Corina I. Megyesi, Laura Rădulescu, Lavinia P. Drăghia, Alexandra T. Lukinich-Gruia, Cornelia Muntean, Daniel I. Hădărugă, Nicoleta G. Hădărugă

**Affiliations:** 1 Department of Food Science, Banat’s University of Agricultural Sciences and Veterinary Medicine “King Michael I of Romania” from Timişoara, Timişoara, Romania; 2 Department of Economics and Company Financing, Banat’s University of Agricultural Sciences and Veterinary Medicine “King Michael I of Romania” from Timişoara, Timişoara, Romania; 3 Centre for Gene and Cellular Therapies in the Treatment of Cancer–OncoGen, Clinical County Hospital of Timişoara, Timişoara, Romania; 4 Department of Applied Chemistry and Engineering of Inorganic Compounds and Environment, Polytechnic University of Timişoara, Timişoara, Romania; 5 Research Institute for Renewable Energy, Polytechnic University of Timişoara, Timișoara, Romania; 6 Department of Applied Chemistry, Organic and Natural Compounds Engineering, Polytechnic University of Timişoara, Timişoara, Romania; Universidade do Porto, Faculdade de Farmácia, PORTUGAL

## Abstract

The goal of the present study was the evaluation of the fatty acid (FA) profile of lipid fraction from dry common beans (*Phaseolus vulgaris* L.) (CBO) harvested from North-East (NE) and South-West (SW) of Romania and to protect against thermal and oxidative degradation of the contained omega-3 and omega-6 polyunsaturated fatty acid (PUFA) glycerides by β-cyclodextrin (β-CD) nanoencapsulation, using kneading method. The most abundant FAs in the CBO samples were PUFAs, according to gas chromatography-mass spectrometry (GC-MS) analysis. Linoleic acid (methyl ester) was the main constituent, having relative concentrations of 43.4 (±1.95) % and 35.23 (±0.68) % for the lipid fractions separated from the common beans harvested from the NE and SW of Romania, respectively. Higher relative concentrations were obtained for the omega-3 α-linolenic acid methyl ester at values of 13.13 (±0.59) % and 15.72 (±0.30) % for NE and SW Romanian samples, respectively. The omega-3/omega-6 ratio consistently exceeds the lower limit value of 0.2, from where the PUFA glyceride mixture is valuable for the human health. This value was 0.32 (±0.02) for the NE samples and significantly higher for the CBO-SW samples, 0.51 (±0.01). These highly hydrophobic mixtures especially consisting of PUFA triglycerides provide β-CD complexes having higher thermal and oxidative stability. Kneading method allowed obtaining β-CD/CBO powder-like complexes with higher recovery yields of >70%. Thermal analyses of complexes revealed a lower content of hydration water (3.3–5.8% up to 110°C in thermogravimetry (TG) analysis and 154–347 J/g endothermal effect in differential scanning calorimetry (DSC) analysis) in comparison with the β-CD hydrate (12.1% and 479.5–480 J/g, respectively). These findings support the molecular inclusion process of FA moieties into the β-CD cavity. Attenuated total reflectance-Fourier transform infrared spectroscopy (ATR-FTIR) analysis reveals the formation of the β-CD/CBO inclusion complexes by restricting the vibration and bending of some bonds from the host and guest molecules. Moreover, powder X-ray diffractometry (PXRD) analysis confirm the formation of the host-guest complexes by modifying the diffractograms for β-CD/CBO complexes in comparison with the β-CD and β-CD + CBO physical mixtures. A significant reduction of the level of crystallinity from 93.3 (±5.3) % for β-CD to 60–60.9% for the corresponding β-CD/CBO complexes have been determined. The encapsulation efficiency (EE), the profile of FAs, as well as the controlled release of the encapsulated oil have also been evaluated. The EE was >40% in all cases, the highest value being obtained for β-CD/CBO-SW complex. The SFA content increased, while the unsaturated FA glycerides had lower relative concentrations in the encapsulated CBO samples. It can be emphasized that the main omega-3 FA (namely α-linolenic acid glycerides) had close concentrations in the encapsulated and raw CBOs (13.13 (±0.59) % and 14.04 (±1.54) % for non-encapsulated and encapsulated CBO-NE samples, 15.72 (±0.30) % and 12.41 (±1.95) % for the corresponding CBO-SW samples, respectively). The overall unsaturated FA content significantly decreased after complexation (from 19.03–19.16% for the raw CBOs to 17.3–17.7% for encapsulated oils in the case of MUFAs, and from 55.7–58.8% to 35.13–43.36% for PUFAs). On the other hand, the omega-3/omega-6 ratio increased by β-CD nanoencapsulation to 0.51 (±0.07) and 0.76 (0.26) for β-CD/CBO-NE and β-CD/CBO-SW complexes, respectively. As a conclusion, the lipid fractions of the Romanian common beans are good candidates for β-CD complexation and they can be protected against thermal and oxidative degradation in common beans based food products such as functional foods or food supplements using natural CDs.

## Introduction

The use of healthy food products is a continuous challenge. Food matrix is a very complex mixture of components that interacts each other and with the environment. Air is the most common environmental factor that interact with the food components from the processing to the storage and consuming levels. During these steps, some thermal and oxidative labile components can be degraded to harmful derivatives. It is the case of lipids, especially those consisting of higher concentrations of polyunsaturated fatty acid (PUFA) glycerides [1]. They are easily degraded to hydroperoxides and free radicals as intermediates, which are harmful for the human health. Further, aldehydes are the main final degradation compounds that negatively affect the overall flavor of the food products.

Common bean (*Phaseolus vulgaris* L.) belongs to Fabaceae botanical family. It is one of the most consumed herbaceous plant both as dried (harvested at complete maturity) or unripe fruits [2–4]. Among other constituents such as starch, protein, dietary fiber, iron, potassium, selenium, molybdenum, thiamine, vitamin B_6_, and folate [5], dry common beans contain up to 2.5% lipid fraction [6, 7]. Even the most concentrated fatty acids are oleic and linoleic acids, common beans lipid fractions contain important parts of α-linolenic acid (as glycerides) [8–10]. This omega-3 fatty acid has a high level of unsaturation. Consequently, α-linolenic acid containing glycerides can be easily oxidized. The protection of these types of compounds against oxidation can be performed by various methods, including micro- and nanoencapsulation. Lipids are generally used as shell materials for microencapsulation. However, bioactive omega-3 based lipids are often subjected to micro- and nanoencapsulation (as core or guest compounds) in order to protect them against oxidative degradation and to reduce the unpleasant odor (such as in the case of fish oils). There are various micro- and nanoencapsulation materials and techniques designed for lipids and lipophilic bioactive compounds. Nanoemulsions are widely used for these purposes [11–13]. They are using low or high molecular weight surfactants and biopolymers (e.g., proteins, starches, pectins), as well as sugars and maltodextrins as coencapsulants for omega-3 based lipids. However, neither nanoemulsions nor liposomes can provide an intimate interaction between the core and shell materials that could prevent oxygen access to the reactive sites of the labile glycerides. On the other hand, non-natural and/or chemically modified components are often used for obtaining such materials [11]. The molecular encapsulation allows obtaining host-guest molecular inclusion complexes having better oxidative stability, such as in the case of natural cyclodextrins (CDs) [14].

CDs are good candidates for protecting hydrophobic and easily oxidizable molecules such as polyunsaturated triglycerides, due to their fatty acid moieties. CDs are natural cyclic oligosaccharides having structures like truncated cones [14, 15]. They consist of six, seven or eight α(1→4) linked glucopyranose moieties corresponding to α-, β- and γ-CD. Due to the obtaining by enzymatic synthesis from starch, using *Bacillus macerans* or cyclodextrin-glucosyl-transferase, only these three natural CDs are FDA/GRAS accepted for using in various food products as well as pharmaceutical formulations [15]. However, chemically modified CDs are only approved for drug nanoencapsulation, but not for food products. CDs advantages consist of protecting against oxidation / degradation and controlled release of hydrophobic and geometrically compatible compounds. The encapsulation provides an increased apparent water solubility and bioavailability of biologically active compounds. Moreover, CD nanoencapsulation allows obtaining powdery complexes containing oily compounds. As a consequence, it reduces the undesirable smell and taste of “off-flavoring” compounds [16–18]. These properties are especially due to the presence of a high number of primary and secondary hydroxyl groups and a hydrophobic cavity for the CD structure. This chemical architecture allows the formation of a host-guest molecular inclusion complex by nanoencapsulation [19]. There are some studies related to the nanoencapsulation of oils and fats by CDs. CD nanoencapsulation was especially used for fish and vegetable oils. The performance of the formation of the host-guest inclusion compounds was revealed by thermal, spectroscopic, X-ray diffractometric, and electron microscopic techniques [17, 20–24]. Various methods are used for CD nanoencapsulation, such as crystallization from alcohol-water solution, spray-drying, slurry or kneading [14–16]. Due to the difference on the solubility characteristics of the host-guest components (CD is a hydrophilic compound with high water solubility, while lipids are very hydrophobic and water insoluble), the kneading technique is the most appropriate for CD-lipid nanoencapsulation [17, 21, 22]. Moreover, this method allows to attain the equilibrium between the host and guest components due to the intimate interaction during the kneading for a longer time at moderate temperature. To our knowledge, no studies on the CD nanoencapsulation of common beans lipids have been performed.

No literature data on cyclodextrin complexation of beans lipid fractions exists. Moreover, there are only few studies related to controlled release of lipid components from cyclodextrin complexes [25, 26]. They are especially related to longer chain omega-3 fatty acid glycerides such as EPA and DHA [26]. No information on similar studies on vegetable omega-3 based glycerides (lipid fractions containing α-linolenic acid glycerides) were performed until now. As a consequence, the novelty and the significance of the research are highlighted by the protection of omega-3 fatty acid glycerides and similar compounds from common beans lipid fractions by β-cyclodextrin nanoencapsulation, the characterization and evaluation of these complexes in order to use this information for development of new food supplements or functional food products based on vegetable omega-3 fatty acids.

The purpose of the study was the evaluation of the fatty acid (FA) profile of the lipid fraction of dry common beans that are growing in different regions of Romania and the lipid interaction with food grade β-CD, the cheapest, FDA/GRAS approved and the most available among natural CDs. This is the first attempt to nanoencapsulate common beans lipid components by CDs in order to evaluate the possibility to use them for protecting the thermal and oxidative labile components in Romanian common beans (*Phaseolus vulgaris* L.) based food products.

## Materials and method

### Materials

Common beans (*Phaseolus vulgaris* L.) samples were harvested at complete maturity (named dry common beans) from two representative regions in Romania in the summer of the year 2017. The same autochthonous white variety of common beans cultivated in both the North-East (NE) and South-West (SW) of the country were harvested from the following locations: 46°38′18″N 27°43′45″E for the NE samples and 44°37′24″N 22°40′04″E for the SW samples (samples were obtained from the local markets). Dry common beans samples were stored in a cold, dark and dry space until lipid extraction. β-CD hydrate had a purity of >98% and a water content by loss on drying of 12.4%, according to manufacturer (CycloLab, Budapest, Hungary). Petroleum ether (reagent grade) of boiling temperature range of 40–60°C have been used for common beans oil (CBO) extraction (Sigma-Aldrich, St. Louis, MO, USA). BF_3_·MeOH (20% boron trifluoride, Merck & Co., Inc., Kenilworth, NJ, USA) and hexane (GC-grade, Sigma-Aldrich, St. Louis, MO, USA) were used for derivatization of CBO samples to the corresponding fatty acid methyl esters (FAMEs). Identification of the main FAMEs in the derivatized CBO samples by gas chromatography-mass spectrometry (GC-MS) analysis was performed using both FAME standard mixture (FAME37, Sigma-Aldrich, St. Louis, MO, USA) and FAME retention indices (RIs) based on GC-MS analysis of C_8_-C_20_ alkane standard mixture (Sigma-Aldrich, St. Louis, MO, USA). Previous GC-MS data on the main FAMEs have been used for further identification, especially for palmitic, stearic, arachidic, behenic, oleic, linoleic and α-linolenic acids, which were standard compounds obtained from Sigma-Aldrich (St. Louis, MO, USA) and Fluka Chemie GmbH (Buchs, Switzerland). Other compounds used for extraction and β-CD complexation were ethanol (96%, *v/v*, Chimopar, Bucharest, Romania), sodium chloride (*p*.*a*., Reactivul, Bucharest, Romania) for organic-aqueous layer separation in the derivatization process and anhydrous sodium sulphate (*p*.*a*., Merck & Co., Inc., Kenilworth, NJ, USA) for drying the extracts and derivatized samples.

### Extraction and derivatization of CBO samples

Dry common beans samples from the NE and SW of Romania were ground using a household grinder (Myria MY4053, Romania), sieved using a 16 mesh household sieve (particle diameter <1.18 mm) and extracted using Soxhlet method without other sample preparation steps. A 200 mL extraction apparatus equipped with 1 L one neck round bottom flask and bulb condenser have been used. Ground and sieved common beans of 82.9 (±12.2) g for NE samples and 106.2 (±35.5) g for SW samples have been used for Soxhlet extraction. The extraction flask was fill with 250 mL of petroleum ether. At least ten consecutive extractions were performed for every sample for a complete separation of the lipid fraction. The extracts were then dried over anhydrous sodium sulphate (approximately 2 g for every extract), concentrated in the same apparatus and the solvent traces were completely removed overnight at room temperature (until no ethereal odor could be detected). The CBO samples were hermetically sealed, stored at 4°C until derivatization and β-CD complexation (in order to prevent possible degradations in the presence of air and light). Both CBO-NE and CBO-SW sample types were obtained, derivatized and complexed in duplicate (coded as duplicates “a” and “b”).

The derivatization of CBO samples to the corresponding FAMEs was performed by transesterification using BF_3_·MeOH method [21, 22, 24]. CBO of 103.5 (±6.1) mg for NE samples and 102.0 (±1.6) mg for SW samples were refluxed with 5 mL of BF_3_·MeOH reagent for 45 minutes in a 100 mL round bottom flask equipped with reflux condenser. The mixture was refluxed for another 15 minutes in the presence of 5 mL hexane. After cooling, the derivatized solution was transferred into a flask having thin neck, containing approximately 15 mL of saturated sodium chloride solution. The mixture was completed with the same salt solution until the organic layer was separated in the neck part. The derivatized hexane solution was transferred into a vial containing ~0.1 g anhydrous sodium sulphate, using a Pasteur pipette. The hexane solution was stored at 4°C until GC-MS analysis.

### Gas chromatography-mass spectrometry (GC-MS) analysis of the derivatized CBO samples

The derivatized CBO samples to the corresponding FAMEs were analyzed by gas chromatography-mass spectrometry (GC-MS). A Hewlett Packard 6890 Series GC coupled with a Hewlett Packard 5973 Mass Selective Detector have been used (licensed for Centre for Gene and Cellular Therapies in the Treatment of Cancer–OncoGen, Clinical County Hospital of Timişoara, Romania; no permission was required because authors that performed the work are employees of the authority). The GC column was a Zebron 5-MS (length 30 m, inner diameter 0.25 mm, film thickness 0.25 μm) and the temperature program was from 50°C to 300°C (6°C/min). Other conditions were: injector temperature 300°C, detector temperature 300°C, carrier gas–Helium, injection volume 2 μL, solvent delay 7 minutes. The following MS setting was used: EI energy 70 eV, source temperature 150°C, scan range 50–300 amu, scan rate 1 / s.

### Synthesis of β-cyclodextrin / common beans oil complexes (β-CD/CBO)

The complexation method was kneading due to the significant differences between the hydrophobicity of host and guest molecules. Consequently, the kneading was performed starting at moderate temperature of 50°C in a ceramic mortar. Thus, 661.5 (±0.7) mg of β-CD hydrate and 464.5 (±0.7) mg of CBO-NE, as well as 667.0 (±4.2) mg of β-CD hydrate and 474.0 (±2.8) mg of CBO-SW, at an approximately molar ratio of 1:1 (taking into account the mean molar mass of β-CD hydrate containing 12.4% water as 1322.1 g/mole and the mean molar mass of CBO, according to GC-MS analysis, of approximately 863 g/mole) have been used. β-CD was solubilized in 2 mL warm water in the mortar and then the mixture of CBO in 1 mL of ethanol was added during a continuous grinding. The kneading was performed for 60 minutes and the complex was stored overnight in a dark place at room temperature for drying. The dried complex was then ground to obtain a fine powder. The final product was sealed and stored at 4°C until further analyses.

### Thermogravimetry (TG) and differential scanning calorimetry (DSC) of β-CD/CBO complexes

Thermal analyses of β-CD/CBO complexes were performed by thermogravimetry-differential thermogravimetry (TG-DTG) and differential scanning calorimetry (DSC). A Netzsch TG 209F1 Libra equipment (Netzsch Group, Selb, Germany, licensed for Polytechnic University of Timişoara, Romania; no permission was required because authors that performed the work are employees of the authority) have been used. The TG-DTG parameters were: heating temperature from 25 to 500°C (heating rate of 10°C/min), dynamic purge flow of 20 mL/min, protective flow of 40 mL/min. For the DSC analysis a Netzsch DSC 204 F1 Phoenix® (Netzsch Group, Selb, Germany, licensed for Polytechnic University of Timişoara, Romania; no permission was required because authors that performed the work are employees of the authority), working with similar conditions have been used (heating temperature from 25 to 490°C, with a heating rate of 10°C/min, dynamic purge and protective flows of 50 mL/min). The acquisition and handling of TG-DTG and DSC data were performed using Proteus® Software for Thermal Analysis ver. 6.1.0 (Netzsch Group, Selb, Germany, licensed for Polytechnic University of Timişoara, Romania; no permission was required because authors that performed the work are employees of the authority).

### Attenuated total reflectance–Fourier transform infrared spectroscopy (ATR-FTIR) analysis

A Bruker Vertex 70 FTIR equipment (Bruker Optik GmbH, Ettlingen, Germany, licensed for Polytechnic University of Timişoara, Romania; no permission was required because authors that performed the work are employees of the authority) equipped with a DLaTGS detector (deuterated lanthanum α-alanine doped triglycine sulphate) and a single-reflection Platinum diamond attenuated total reflectance sampling module, ATR, have been used for ATR-FTIR analysis. The apparatus have a spectral range of 12000–250 cm^-1^ and a sensitivity of D* > 2×10^8^ cm Hz^1/2^ W^-1^. The following parameters were used for ATR-FTIR analysis of both liquid (CBO samples) and solid samples (β-CD and β-CD/CBO complexes): acquisition range 4000–400 cm^-1^, sensitivity >0.5%, number of sample scans 128, resolution 4 cm^-1^, apodization function—Blackman-Harris 3-Term, phase correction mode—power spectrum, phase resolution 32, air background spectra (before every analysis), mass of the sample for analysis (liquid or solid) of 10–20 mg, software for acquisition and handling of the ATR-FTIR data—OPUS ver. 7.2 (Bruker Optik GmbH 2012, Ettlingen, Germany, licensed for Polytechnic University of Timişoara, Romania; no permission was required because authors that performed the work are employees of the authority).

### Powder X-ray diffractometry (PXRD) analysis

A Rigaku Ultima IV X-ray diffractometer (Rigaku, Tokyo, Japan, licensed for Research Institute for Renewable Energy, Polytechnic University of Timişoara, Romania; no permission was required because authors that performed the work are employees of the authority) have been used for powder X-ray diffractometry (PXRD) analysis of β-CD hydrate, β-CD/CBO complexes, as well as β-CD and CBO physical mixtures. The following parameters were used for PXRD analysis: a Cu Kα radiation (λ = 1.54060 Å) at 40 kV and 40 mA, scanning range 2θ of 10.000–59.990°, step size 0.010°, 96 s per scan. The equipment has the possibility to automatic alignment of tube height, goniometer, optics and detector. The PDXL 2 software (Rigaku, Tokyo, Japan, licensed for Research Institute for Renewable Energy, Polytechnic University of Timişoara, Romania; no permission was required because authors that performed the work are employees of the authority) have been used for the acquisition of the PXRD data. The handling of the PXRD data was performed with the WinPLOTR software (July 2017, T. Roisnel & J. Rodriguez-Carvajal, UMR-CNRS 6226 Rennes & ILL Grenoble; no permission was required for academic research, according to above-mentioned owners; citing the references is indicated [27, 28]).

### Encapsulation efficiency of common beans oil in β-CD/CBO complexes

Encapsulation efficiency (EE) was determined according to [26] with slight modifications for CBO samples. Shortly, 0.056 (±0.001) g of β-CD/CBO complexes were mixed with 5 mL hexane for one minute in order to solubilizes the surface oil (non-encapsulated CBO samples). The solution was filtered, evaporated to dryness and weighted. Surface CBO mass have been obtained (grams). The encapsulated oil was separated from the superficially washed β-CD/CBO complexes using the same procedures. Three subsequent extractions and separations using 5 mL hexane for every extraction for 15 minutes at room temperature have been performed. The united extracts were evaporated to dryness and the encapsulated oil was weighted (Encapsulated CBO mass, grams). Total CBO content was determined as a sum of surface and encapsulated CBO. The EE was determined according to the following equation:

EE(%)=EncapsulatedCBO(g)/[SurfaceCBO(g)+EncapsulatedCBO(g)]×100

Both β-CD/CBO-NE and β-CD/CBO-SW complex samples have been analyzed as duplicates.

### Fatty acid profile of encapsulated common beans oils

Encapsulated CBO samples (separated by hexane multiple extraction after removing the surface oil from the β-CD/CBO complexes) were evaluated for their fatty acid profile. Consequently, the recovered CBO samples were dissolved with 5 mL hexane and subjected to derivatization to the FAMEs using the above-mentioned MeOH·BF_3_ method. The hexane FAME solution was dried over anhydrous sodium sulphate and analyzed by GC-MS, using the same conditions such as for the raw CBO samples. All determinations were made as duplicates.

### Controlled release of the encapsulated CBO from β-CD/CBO complexes

The evaluation of the release of encapsulated oil from cyclodextrin complexes is very difficult in such systems. However, 0.9% NaCl aqueous solution (which simulate the salt solutions for perfusions) and hexane layer (simulating the hydrophobic parts in the human body, such as cell membranes) have been used for controlled release of encapsulated CBO from the complexes [26]. Thus, 0.053 (±0.004) g of β-CD/CBO complex was dissolved in 20 mL of 0.9% NaCl solution and 20 mL of hexane was used as the upper layer. The heterogeneous system was vigorously mixed at room temperature using a magnetic stirring system (750 rpm, Heidolph MR Hei-Standard, Heidolph Instruments, Schwabach, Germany). The system was hermetically sealed and samples of 1 mL at 30, 60, 120 and 240 min have been taken off from the upper layer and evaporated to dryness at room temperature in the dark using aluminum crucibles. The hexane layer was completed with the same solvent. The separated and dry oil was weighed for every sample and the corrected total mass was converted to percent of the CBO released, knowing the total oil encapsulated from the EE data. The actual CBO release (%) was plotted against time (h). Two measurements were performed for every β-CD/CBO complex.

### Statistical analysis

Classical ANOVA (ANalysis Of VAriance) have been used for statistical evaluation of all data obtained by complexation (yields), encapsulation efficiency, controlled release and physical chemical analyses (GC-MS, TG-DTG, DSC, ATR-FTIR, PXRD) of β-CD hydrate, CBO samples, β-CD/CBO complexes, β-CD and CBO physical mixtures, as well as statistical evaluation of PXRD signals for calculating the crystallinity index. All results were provided as the average and standard deviation (SD) for multiplicate analyses. The Microsoft® Excel ® 2013, Microsoft Office Professional Plus 2013 (Microsoft Co., licensed for Polytechnic University of Timişoara, Romania; no permission was required because authors that performed the work are employees of the authority) have been used for the above-mentioned statistics.

## Results and discussion

### Fatty acid profile of CBO samples

CBO samples were obtained by solid-liquid semi-continuous Soxhlet extraction using at least ten successive extractions for a complete recovering of the lipid fractions. Control sample for the last extracts did not reveal any lipid residue after evaporation. Consequently, it was assumed that the lipid fractions were completely recovered from the finely ground common beans samples. The overall recovery yield was determined as the percent ratio between the total mass of the combined dry CBO fractions and the mass of the starting ground common beans. The extraction yield for CBO-NE samples was 2.25 (±0.65) %, while for CBO-SW sample this yield was little bit lower, 1.65 (±0.13) %.

The FA profile of both CBO-NE and CBO-SW sample types was evaluated after derivatization to the corresponding FAMEs, which are more volatile and appropriate to be analyzed by GC-MS. The identification of the main compounds was performed using both MS identification by comparison of the actual MS spectra with those from the NIST/EPA/NIH Mass Spectral Database (NIST 11) using the NIST MS Search 2.0g program (NIST, Gaithersburg, MD, USA, licensed for Centre for Gene and Cellular Therapies in the Treatment of Cancer–OncoGen, Clinical County Hospital of Timişoara, Romania; no permission was required because authors that performed the work are employees of the authority), and by means of the retention index (RI) values, determined using the C_8_-C_20_ alkane standard mixture and the RI values for standard FAMEs (determined in the same conditions in previously reported studies [21, 22, 24]). All GC chromatograms and MS spectra for the main FAMEs (both experimental and from the NIST database) are presented in Figures A-D in S1 File for GC and Figures E-S in S1 File for MS spectra. The relative concentrations of FAMEs and some degradation compounds (such as aldehydes, identified as their dimethyl acetals) were determined as the percent ratio between the corresponding peak area and the sum of peak area for all separated compounds. The identified compounds, their codes according to the FA class (or other chemical classes), the RI values (including the retention time, RT, min, in parenthesis), as well as the relative concentration (both as mean ± SD and concentration interval, in parenthesis) of the FAMEs and other degradation compounds identified in CBO samples are presented in Table 1. By far, the most concentrated FAs (as methyl esters) in the CBO samples were PUFAs. Among these, the omega-3 FA named α-linolenic acid had relative concentrations of 13.13 (±0.59) % in CBO-NE samples and slightly higher in CBO-SW samples, 15.72 (±0.30) % (Table 1). The other important PUFA was linoleic acid, having the highest concentration in both sample types: 43.40 (±1.95) % and 35.23 (±0.68) % for CBO-NE and CBO-SW samples, respectively. These FAs were also identified as monoglycerides (probably due to an incomplete transesterification of the corresponding triglycerides from the CBO samples), such as 1-mono-α-linolenin (Table 1), which were also quantified as PUFA. On the other hand, monounsaturated fatty acids (MUFAs) and saturated fatty acids (SFAs) were identified in very close concentrations of 19.03–19.16% and 20.18–22.84%, respectively (Table 1). Among MUFAs, the most concentrated was oleic acid (as methyl ester), having a relative concentration of 17.41 (±1.11) % in the CBO-NE samples and slightly lower in the CBO-SW samples, 16.68 (±0.08) %. It must be pointed out that oleic acid can be partially isomerized to the *trans* isomer, elaidic acid, which was identified at a RI close to that of oleic acid, and was difficult to identify. The MS spectra was similar with that for the other *cis* isomer of oleic acid, the vaccenic acid (as methyl esters). However, a significant GC peak appear at RI of 2107.1, having relative area up to 1.61% in both CBO types, which was attributed to the above-mentioned compounds (Table 1). Other MUFAs were petroselinic acid (<0.17%), palmitoleic acid (<0.53%), and even gondoic acid (C20:1(11*c*) at a concentration of the methyl ester of 0.17–0.25%, Table 1). The number of SFAs was significantly higher, even the overall concentration was low in comparison with PUFAs. At least twelve SFAs have been identified in both CBO-NE and CBO-SW sample types. Among these, palmitic acid was the most concentrated (as methyl ester at relative concentrations of 13.90 (±1.80) % and 17.36 (±0.41) % for CBO-NE and CBO-SW samples, respectively, Table 1). Stearic acid also had significant concentration up to 3.32%, being slightly higher in the CBO-NE samples. Concentrations lower than 0.77% were determined for caproic, pelargonic, myristic, pentadecanoic, margaric, arachidic, heneicosanoic, behenic, tricosanoic, and lignoceric acids (Table 1). Similar FA distribution in the common beans lipid fractions have been obtained by other research groups for other varieties of common beans. They identified higher α-linolenic acid concentrations in such samples [7, 9, 10]. They reported up to 46% α-linolenic acid (as methyl ester) for autochthonous Slovenian *Phaseolus vulgaris* species [7], which is higher than our findings. On the other hand, Yoshida and co-workers demonstrate that the FAs are mainly bound as triacylglycerols in *Phaseolus vulgaris* species from Japan, the main FA being α-linolenic acid [9, 10].

**Table 1:** Gas chromatography-mass spectrometry (GC-MS) data for the derivatized common bean lipid fractions (*Phaseolus vulgaris* L.) from the NE and SW of Romania (raw and encapsulated CBO-NE and CBO-SW, respectively).

N^o^	MS Identification^a^	Code / ω class	*RI* (*RT*, min)	*Area* (%) ± SD (min-max, %) CBO-NE	*Area* (%) ± SD (min-max, %) CBO-SW	*Area* (%) ± SD (min-max, %) CBO-NE encapsulated	*Area* (%) ± SD (min-max, %) CBO-SW encapsulated
1	Caproic acid, methyl ester	C6:0	922.3 (4.002)	0.01 (0.00–0.02)	-	-	-
2	Hexanal, dimethyl acetal	Ald	975.0 (5.016)	0.01 (0.00–0.01)	0.05 ± 0.03 (0.03–0.07)	-	-
3	Pelargonic acid, methyl ester	C9:0	1225.7 (10.480)	0.06 (0.01–0.11)	0.01 ± 0.00 (0.01–0.01)	0.06 (0.00–0.06)	-
4	Nonanal, dimethyl acetal	Ald	1278.0 (11.668)	(0.00–0.01)	0.02 ± 0.01 (0.01–0.02)	-	-
5	Suberic acid, dimethyl ester	DiAcid	1443.6 (15.286)	0.01 (0.00–0.01)	0.01 (0.00–0.01)	-	-
6	Azelaic acid, dimethyl ester	DiAcid	1544.4 (17.332)	0.03 (0.01–0.06)	0.04 ± 0.02 (0.02–0.06)	-	-
7	Myristic acid, methyl ester	C14:0	1727.1 (20.744)	0.25 ± 0.00 (0.24–0.25)	0.25 ± 0.00 (0.24–0.25)	0.54 ± 0.13 (0.44–0.63)	0.8 (0.00–0.80)
8	Petroselinic acid, methyl ester	C16:1(6c)/omega-12	1811.8 (22.225)	0.07 ± 0.01 (0.07–0.08)	0.17 ± 0.00 (0.17–0.17)	-	-
9	Pentadecanoic acid, methyl ester	C15:0	1828.8 (22.516)	0.14 ± 0.02 (0.13–0.16)	0.23 ± 0.00 (0.23–0.23)	0.31 ± 0.13 (0.23–0.40)	0.32 (0.00–0.32)
10	Palmitoleic acid, methyl ester	C16:1(9c)/omega-7	1906.7 (23.831)	0.47 ± 0.08 (0.42–0.53)	0.43 ± 0.02 (0.41–0.44)	0.47 ± 0.19 (0.33–0.60)	0.53 (0.00–0.53)
11	**Palmitic acid, methyl ester**	C16:0	1933.9 (24.282)	**13.90 ± 1.80** (12.63–15.17)	**17.36 ± 0.41** (17.07–17.65)	**17.41 ± 3.74** (14.77–20.06)	**18.71 ± 6.64** (14.01–23.4)
12	Margaric acid methyl ester	C17:0	2027.7 (25.826)	0.19 ± 0.01 (0.19–0.20)	0.23 ± 0.00 (0.23–0.23)	0.24 ± 0.02 (0.22–0.25)	0.22 (0.00–0.22)
13	**Linoleic acid, methyl ester**	C18:2(9c,12c)/omega-6	2097.6 (26.969)	**43.4 ± 1.95** (42.02–44.78)	**35.23 ± 0.68** (34.75–35.72)	**28.96 ± 6.28** (24.52–33.4)	**20.34 ± 5.19** (16.67–24.01)
14	**α-Linolenic acid, methyl ester**	C18:3(9c,12c,15c)/omega-3	2103.2 (27.060)	**13.13 ± 0.59** (12.71–13.54)	**15.72 ± 0.30** (15.51–15.93)	**14.04 ± 1.54** (12.95–15.13)	**12.41 ± 1.95** (11.03–13.79)
15	**Oleic acid, methyl ester**	C18:1(9c)/omega-9	2105.3 (27.094)	**17.41 ± 1.11** (16.62–18.19)	**16.68 ± 0.08** (16.62–16.74)	**15.35 ± 1.96** (13.96–16.73)	**15.47 ± 3.00** (13.35–17.6)
16	Elaidic acid, methyl ester / Vaccenic acid, methyl ester*	C18:1(9t/11t)/omega-9/7	2107.1 (27.123)	0.86 (0.12–1.61)	1.43 ± 0.25 (1.25–1.60)	1.49 ± 0.28 (1.29–1.69)	1.95 ± 0.67 (1.47–2.42)
17	16-Octadecenoic acid, methyl ester	C18:1(16t)/omega-2	2113.7 (27.232)	0.04 (0.00–0.09)	0.08 ± 0.00 (0.08–0.09)	-	-
18	**Stearic acid, methyl ester**	C18:0	2123.8 (27.397)	**3.23 ± 0.14** (3.13–3.32)	**2.46 ± 0.10** (2.39–2.53)	**3.4 ± 1.19** (2.55–4.24)	**3.06 ± 1.04** (2.32–3.8)
19	Palmitin, 1,2-di-*	C16:0 (DG)	2268.4 (29.798)	0.18 ± 0.13 (0.09–0.27)	0.52 ± 0.14 (0.42–0.61)	0.86 (0.00–0.86)	3.26 (0.00–3.26)
20	11-Eicosenoic acid, methyl ester (gondoic acid, methyl ester)	C20:1(11c)/omega-9	2273.1 (29.878)	0.22 ± 0.04 (0.20–0.25)	0.18 ± 0.01 (0.17–0.18)	-	-
21	Arachidic acid, methyl ester	C20:0	2296.1 (30.272)	0.7 ± 0.11 (0.62–0.77)	0.44 ± 0.05 (0.41–0.47)	-	-
22	Heneicosanoic acid, methyl ester	C21:0	2372.5 (31.620)	0.06 ± 0.01 (0.05–0.07)	-	-	-
23	9,12-Octadecadienoic acid, (2-phenyl-1,3-dioxolan-4-yl)methyl ester, cis-*	C18:2(9c,12c)/omega-6 (MG deriv.)	2400.0 (32.124)	0.66 ± 0.63 (0.22–1.11)	1.55 ± 0.54 (1.17–1.93)	-	-
24	Linolenin, 1-mono-*	C18:3(9c,12c,15c)/omega-3 (MG)	2405.2 (32.221)	0.95 (0.25–1.66)	3.23 ± 0.20 (3.09–3.37)	0.73 (0.00–0.73)	4.76 (0.00–4.76)
25	Behenic acid, methyl ester	C22:0	2443.4 (32.953)	0.58 ± 0.05 (0.55–0.62)	0.61 ± 0.03 (0.58–0.63)	-	-
26	Tricosanoic acid, methyl ester	C23:0	2504.9 (34.216)	0.15 ± 0.01 (0.15–0.15)	0.17 ± 0.01 (0.17–0.18)	-	-
27	Lignoceric acid methyl ester	C24:0	2557.9 (35.445)	0.4 ± 0.00 (0.4–0.4)	0.53 ± 0.07 (0.49–0.58)	-	-
	*Other compounds*			2.88 ± 0.76 (2.34–3.42)	2.38 ± 0.33 (2.14–2.61)	16.98 ± 3.53 (14.48–19.48)	18.9 ± 9.82 (11.96–25.84)
	Σ *SFA*			20.18 ± 1.73 (18.96–21.4)	22.84 ± 0.12 (22.75–22.93)	22.35 ± 5.82 (18.24–26.47)	24.71 ± 11.84 (16.33–33.08)
	Σ *MUFA*			19.16 ± 0.13 (19.08–19.25)	19.03 ± 0.32 (18.8–19.26)	17.30 ± 2.04 (15.86–18.75)	17.69 ± 3.30 (15.36–20.02)
	Σ *PUFA*			58.82 ± 1.14 (58.01–59.63)	55.74 ± 0.06 (55.7–55.78)	43.36 ± 7.31 (38.2–48.53)	35.13 ± 3.78 (32.46–37.81)
	Σ ω-3			14.1 ± 0.41 (13.81–14.39)	18.92 ± 0.15 (18.81–19.03)	14.4 ± 1.03 (13.68–15.13)	14.79 ± 1.41 (13.79–15.79)
	Σ ω-6			44.24 ± 1.26 (43.35–45.13)	36.79 ± 0.11 (36.71–36.86)	28.96 ± 6.28 (24.52–33.4)	20.34 ± 5.19 (16.67–24.01)
	Σ ω-9			17.63 ± 1.15 (16.82–18.44)	16.85 ± 0.07 (16.8–16.91)	15.35 ± 1.96 (13.96–16.73)	15.47 ± 3.00 (13.35–17.60)
	ω-3/ω-6			0.32 ± 0.02 (0.31–0.33)	0.51 ± 0.01 (0.51–0.52)	0.51 ± 0.07 (0.45–0.56)	0.76 ± 0.26 (0.57–0.95)

The following codes were used: *Cx*:*y* for the fatty acid methyl esters (FAMEs, *x*–the number of C atoms and *y*–the number of double bonds), *c* and *t*–*cis* and *trans* configuration of the double bonds, *Ald*–aldehyde (degradation compound), *DiAcid*–dicarboxylic acid, dimethyl ester (degradation compound), *MG or DG*–mono- or diglyceride, *RI*–retention index, *RT*–retention time (min), *Area* (%)–relative concentration, based on the GC peak area. Relative concentrations are presented as the mean of two determinations ± standard deviation (*SD*) and as the interval (in parenthesis).

^a^ MS identification was based on the mass spectra from the GC-MS analysis and NIST 2011 database; *SFA*—saturated fatty acid, *MUFA*—monounsaturated fatty acid, *PUFA*—polyunsaturated fatty acid, ω-3/-6/-9 – ω classification of the fatty acids.

* Identified with low probability.

It must be pointed out the presence of various degradation compounds in all CBO samples, even at very low relative concentrations. The most important degradation compounds were aldehydes, which were derivatized to the corresponding dimethyl acetals during the transesterification. No more than 0.07% of hexanal have been determined. On the other hand, traces of nonanal were also identified in both sample types (Table 1). Dicarboxylic acids were identified as dimethyl esters in the derivatized CBO samples. Relative concentrations up to 0.06% have been determined for suberic and azelaic acids (Table 1). All other FAMEs and degradation compounds were identified at very low concentrations and were grouped as “other compounds” in Table 1.

The CBO fractions are important and valuable due to their high content of omega-3 FA glycerides, even the overall lipid content of common beans is not so high. The omega-3 FA content almost reach 19%, especially for CBO-SW samples. The presence of omega-3 FA, especially α-linolenic acid, at high concentrations provides an omega-3/omega-6 ratio of 0.32 (±0.02) for CBO-NE samples and significantly higher for CBO-SW samples, 0.51 (±0.01) (Table 1). It is well known that a value over 0.2 provide omega-3 based lipid fractions having healthy properties for both cardio-vascular and neuronal systems [29]. This ratio is almost double in some of the common beans lipid fractions, which suggest the possibility to have healthy implications of such FAs from these types of vegetables.

### Synthesis of β-CD/CBO complexes

The presence of a high content of PUFAs in common beans made less stable their lipid fractions. The FA moieties can be oxidized (easily at higher temperature) to hydroperoxides and further generating free radicals and various final degradation compounds. Some of them were clearly identified in the derivatized CBO samples as aldehydes and dicarboxylic acid derivatives. In order to stabilize and reduce the level of degradation of such labile compounds (especially for the case of omega-3 FA glycerides) the nanoencapsulation by CD can be performed. The FA moieties of glycerides contained by CBO samples fitted into the β-CD cavity and interact by van der Waals forces that provide highly stable complex. On the other hand, oxygen cannot reach the corresponding site for the degrading reaction and thus the FA moiety is protected against degradation. The oily triglycerides and β-CD hydrate provide powdery complexes having higher “apparent” water solubility and bioavailability, as well as controlled release properties. Moreover, the “off-flavor” smell provided by degradation compounds could be reduced. Due to the fact that CBO is a highly hydrophobic liquid and β-CD hydrate is a hydrophilic and water soluble powdery solid, the most appropriate technique for nanoencapsulation is kneading method [30–33]. The host-guest inclusion complexes are formed at molecular level using small amounts of solvents (water and ethanol). During the grinding, the host-guest equilibrium can be reach after appropriate time. The kneading time for β-CD/CBO complexation was one hour, while the suspension became a homogenous paste that can be dried and ground as fine powder. The recovering yield of β-CD/CBO complexes, calculated as percentage of the mass of the complex divided by the sum of the masses of starting materials, i.e. β-CD hydrate and CBO, were in the range of 70.91 (±2.79) % for β-CD/CBO-SW complexes to 77.76 (±4.31) % for β-CD/CBO-NE complexes. These values are due to the loss of an important part of the water molecules during the nanoencapsulation process. The water molecules from the cavity and between the β-CD molecules in the hydrate are replaced by the more hydrophobic triglycerides contained by CBO fractions.

### Thermal analysis of β-CD/CBO complexes and starting compounds

**Fig 1:**
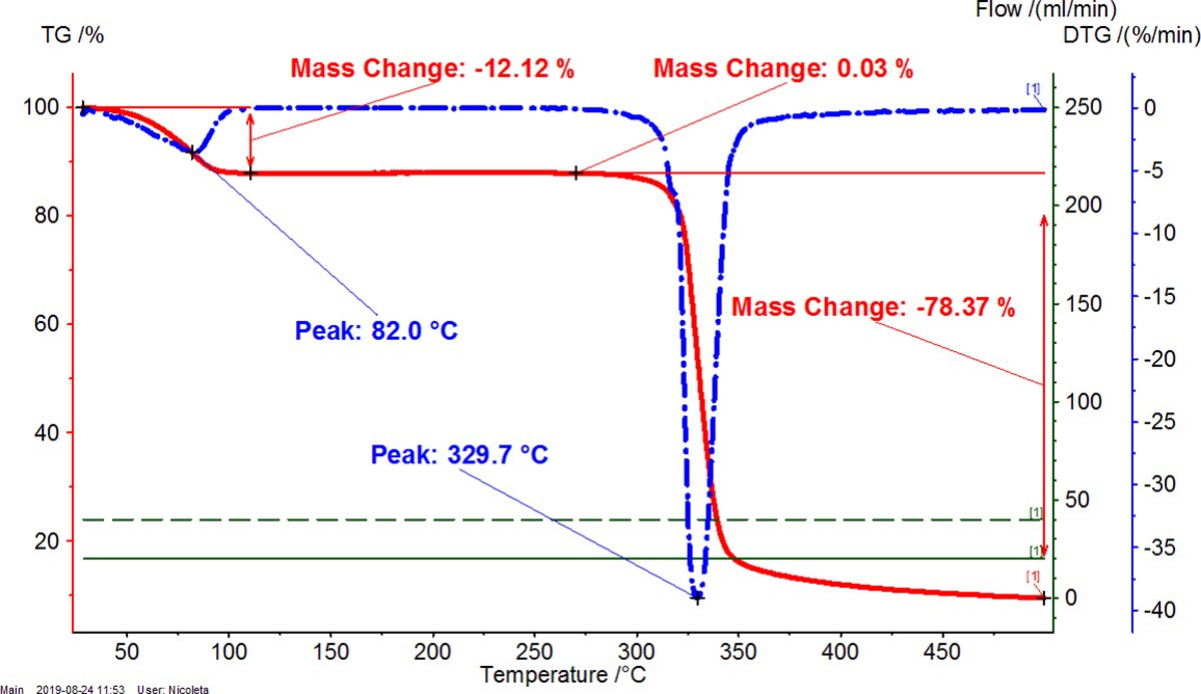
Thermogravimetry-differential thermogravimetry (TG-DTG) analysis of β-cyclodextrin (β-CD) hydrate. The duplicate “a” is presented. The duplicate “b” is presented in Figure X in S1 File.

**Fig 2:**
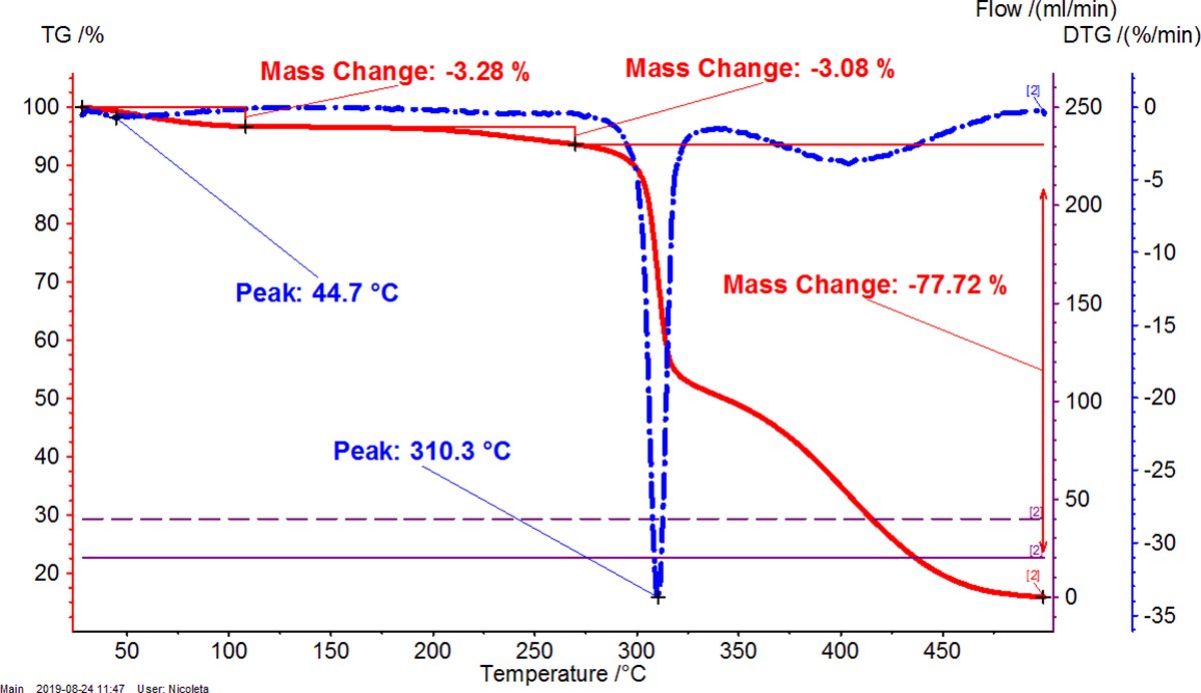
Thermogravimetry-differential thermogravimetry (TG-DTG) analysis of β-CD / CBO-NE (North-East) complex. The duplicate “a” is presented. The duplicate “b” is presented in Figure Y in S1 File.

**Fig 3:**
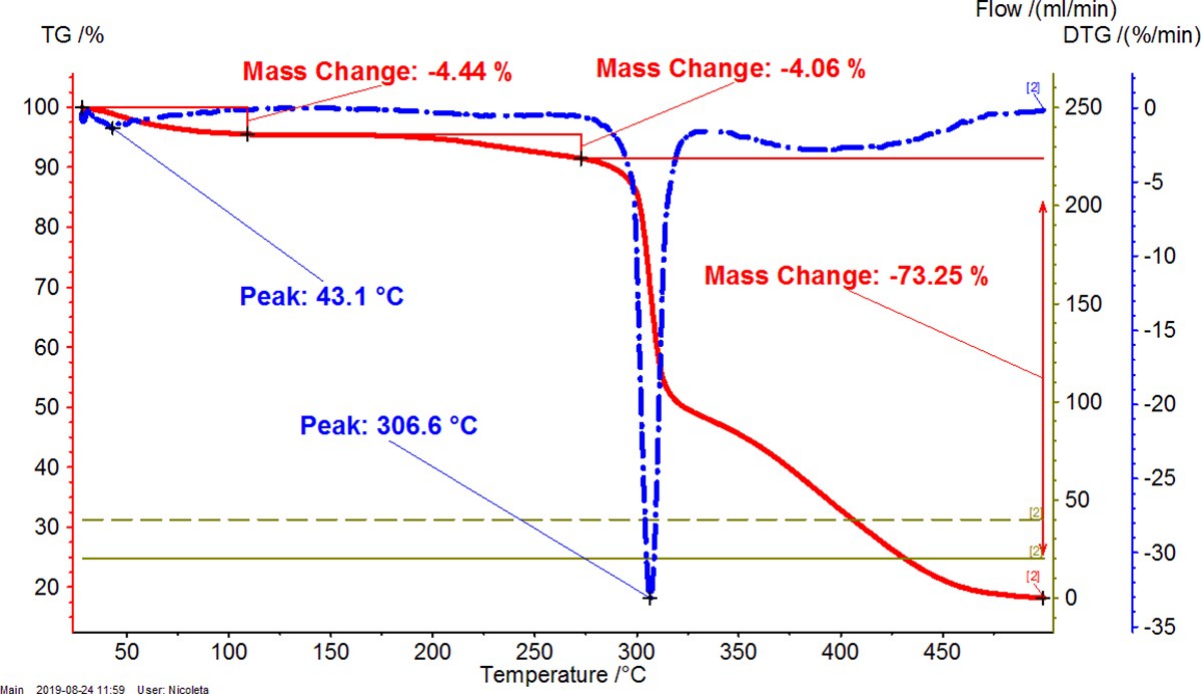
Thermogravimetry-differential thermogravimetry (TG-DTG) analysis of β-CD / CBO-SW (South-West) complex. The duplicate “a” is presented. The duplicate “b” is presented in Figure Z in S1 File.

**Fig 4:**
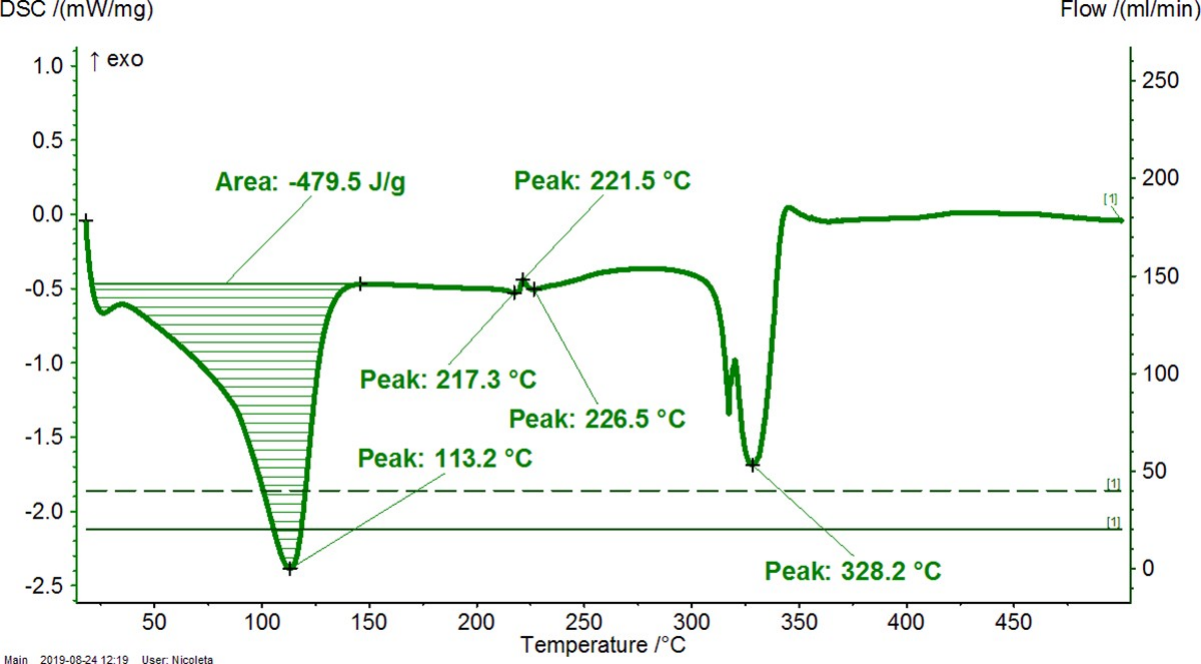
Differential scanning calorimetry (DSC) analysis of β-cyclodextrin (β-CD) hydrate. The duplicate “a” is presented. The duplicate “b” is presented in Figure AA in S1 File.

**Fig 5:**
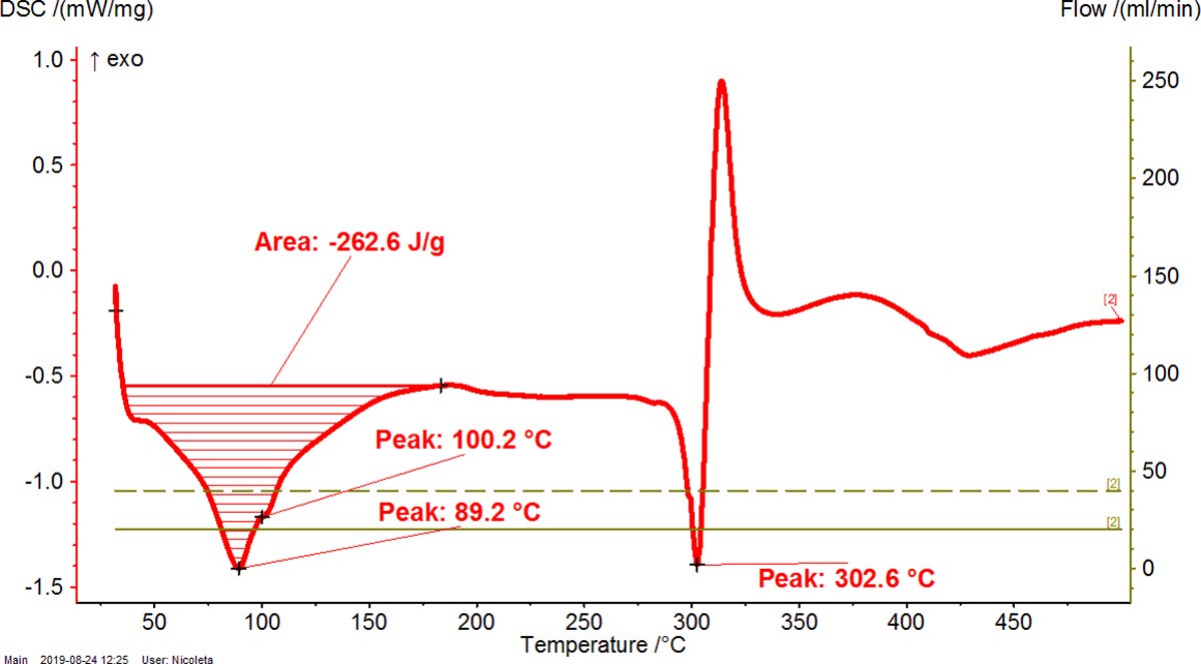
Differential scanning calorimetry (DSC) analysis of β-CD / CBO-NE (North-East) complex. The duplicate “a” is presented. The duplicate “b” is presented in Figure AB in S1 File.

**Fig 6:**
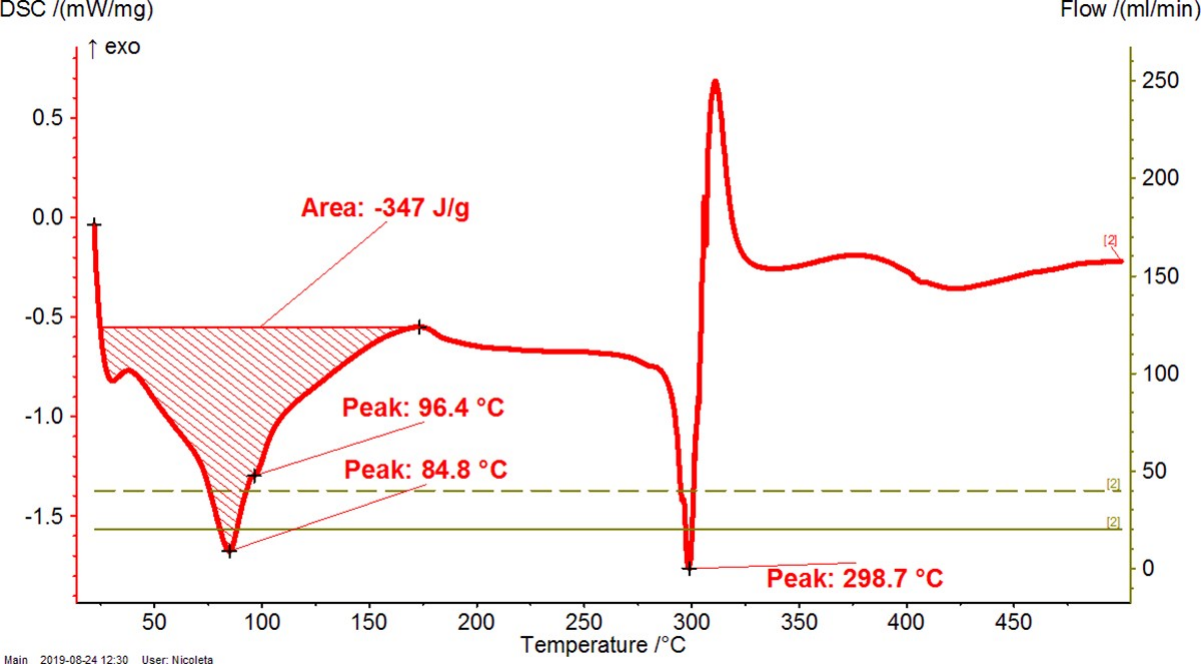
Differential scanning calorimetry (DSC) analysis of β-CD / CBO-SW (South-West) complex. The duplicate “a” is presented. The duplicate “b” is presented in Figure AC in S1 File.

Thermal analyses are appropriate techniques for evaluating the complexation-decomplexation processes, as well as other transitions and even thermal stability. The β-CD/CBO complexes contain many compounds having various properties. β-CD is a cyclic oligosaccharide with high hydrophilicity, which interact with water and other hydrophilic molecules, especially at the exterior of the truncated cone architecture. On the other hand, the inner cavity of β-CD is highly hydrophobic and well encapsulates hydrophobic molecules or moieties such as fatty acid derivatives. CBO contain a complex mixture of such compounds, especially as triglycerides. Consequently, there are more specific temperature ranges during the heating of β-CD/CBO complexes that are related to the dissociation of water molecules (and other solvent molecules such as ethanol), the release of guest compounds (e.g., triglycerides from CBO) and the decomposition of the components of the complex. It was observed for both TG-DTG and DSC analyses that the first temperature range is up to ~110°C, the second one was considered as 110–275°C, and the third corresponds to the start of the decomposition of β-CD and later of the dissociated CBO components (>275°C) (see Figs 1–6, Figures X-Z in S1 File for TG-DTG, Figures AA-AC in S1 File for DSC). Both thermogravimetric and calorimetric curves are significantly different for β-CD/CBO complex in comparison with the starting β-CD hydrate. All ranges have different behavior for complexes. TG-DTG analysis of complexes reveals a mass change corresponding to water (and possible solvent) release up to 110°C of 4.54 (±1.78) % and 5.13 (±0.97) % for β-CD/CBO-NE and β-CD/CBO-SW complexes, respectively (Table 2). On the contrary, the mass change for β-CD hydrate was significantly higher (12.08%). The DTG peak temperatures are also different for this range. This is 43.1–53.7°C for complexes and considerably higher for β-CD hydrate (82.0°C). This aspect support the replacing of a part of water molecules during the molecular encapsulation process. On the other hand, there are especially “surface” water molecules that are released during this temperature interval for β-CD/CBO complexes. However, an important mass change for complexes also appear after 110°C, which can be due to the release of “strongly retained” water molecules, remained in the β-CD cavity and other volatiles (such as ethanol or possible some degradation compounds like aldehydes). Almost no mass change was observed for β-CD during this temperature interval. Starting from ~275°C the decomposition of β-CD begin. There is an important difference between the DTG peak temperature corresponding to the decomposition of starting β-CD and that from the complex. The first decomposes at 329.7°C, while the β-CD from complexes at 301.2–310.3°C (Figs 1–3 and Table 2). The glycerides from CBO start to decompose at ~350°C.

**Table 2:** Thermogravimetry–differential thermogravimetry (TG-DTG) and differential scanning calorimetry (DSC) analysis of β-cyclodextrin/common bean lipid fraction complexes.

N^o^	Code	*ML*_*<110°C*_ (%)	*ML*_*110-275°C*_ (%)	*ML*_*>275°C*_ (%)	*t*_*1(DTG)*_ (°C)	*t*_*2(DTG)*_ (°C)	*A*_*(DSC)*_ (J g^-1^)	*t*_*1(DSC)*_ (°C)	*t*_*2(DSC)*_ (°C)	*t*_*3(DSC)*_ (°C)
1	β-CD	12.08±0.06 (12.04–12.12)	0.03±0.01 (0.02–0.03)	78.35±0.04 (78.32–78.37)	82.0±0.0 (82.0–82.0)	329.7±0.0 (329.7–329.7)	479.9±0.6 (479.5–480.3)	-	113.2±0.0 (113.2–113.2)	328.2±0.0 (328.2–328.2)
2	β-CD/CBO-NE	4.54±1.78 (3.28–5.80)	2.90±0.26 (2.71–3.08)	75.72±2.84 (73.71–77.72)	49.2±6.4 (44.7–53.7)	305.8±6.4 (301.2–310.3)	208.2±76.9 (153.8–262.6)	74.1±21.4 (58.9–89.2)	84.3±22.5 (68.4–100.2)	301.6±1.4 (300.6–302.6)
3	β-CD/CBO-SW	5.13±0.97 (4.44–5.81)	4.26±0.28 (4.06–4.45)	73.09±0.23 (72.92–73.25)	46.6±5.0 (43.1–50.1)	307.6±1.4 (306.6–308.6)	287.5±84.2 (227.9–347)	77.1±11.0 (69.3–84.8)	91.4±7.1 (86.4–96.4)	301.1±3.4 (298.7–303.5)

β-CD/CBO-NE and β-CD/CBO-SW stand for β-cyclodextrin/common bean oil complexes from the North-East and South-West of Romania, respectively. *ML*_*<110°C*_, *ML*_*110-275°C*_, *ML*_*>275°C*_ represent the TG mass loss (%) up to 110°C, in the temperature range of 110–275°C and after 275°C; *t*_*1(DTG)*_ and *t*_*2(DTG)*_ stands for the DTG temperature of the peak (°C) corresponding to water release and decomposition of compounds, respectively; *A*_*(DSC)*_ stands for the DSC peak area corresponding to endothermic process of the water release and degradation of compounds, J/g. *t*_*1-3(DSC)*_ represent the DSC peak temperatures (°C) corresponding to “surface” and “strongly-retained” water release and decomposition of compounds, respectively. Thermal results are presented as the mean ± standard deviation (SD) and intervals (in parenthesis).

DSC analysis of β-CD/CBO complexes and the starting β-CD hydrate reveals similar behavior with an exception related to the transition process from crystalline to amorphous state for anhydrous β-CD at 221.5°C. This endothermal-exothermal effect that take place from 217.3 to 226.5°C (Figs 4–6) disappears in all β-CD/CBO complexes. This is a confirmation that the molecular inclusion process take place and the crystal structure of β-CD is broken after nanoencapsulation [34–36]. The endothermal effect of the dissociation of water molecules is much lower for complexes in comparison with that for β-CD hydrate (153.8–347.0 J/g and 479.9 J/g, respectively, Figs 4–6 and Table 2). Moreover, the DSC peak temperatures appear at lower values than for β-CD (58.9–100.2°C and 113.2°C, respectively). It is a similar behavior for decomposition of β-CD in complexes and as starting material. The DSC peak temperatures appear at 298.7–303.5°C and 328.2°C, respectively (Figs 4–6 and Table 2). Consequently, thermal analyses of β-CD/CBO complexes reveal the formation of the host-guest supramolecular systems by means of replacing the water molecules inside the β-CD cavity by triglycerides contained by CBO samples and through the disappearance of the endo-exothermal effect corresponding to the crystalline-amorphous transition of β-CD.

### ATR-FTIR analysis of β-CD/CBO complexes and starting compounds

ATR-FTIR technique can provide information related to the presence of some compounds having specific bonds or groups, which have stretching or bending vibrations. The FTIR bands can be affected by β-CD molecular inclusion due to the restriction of these vibrations into the host-guest complex. Both the position and intensity of a FTIR band can be affected by nanoencapsulation.

**Fig 7:**
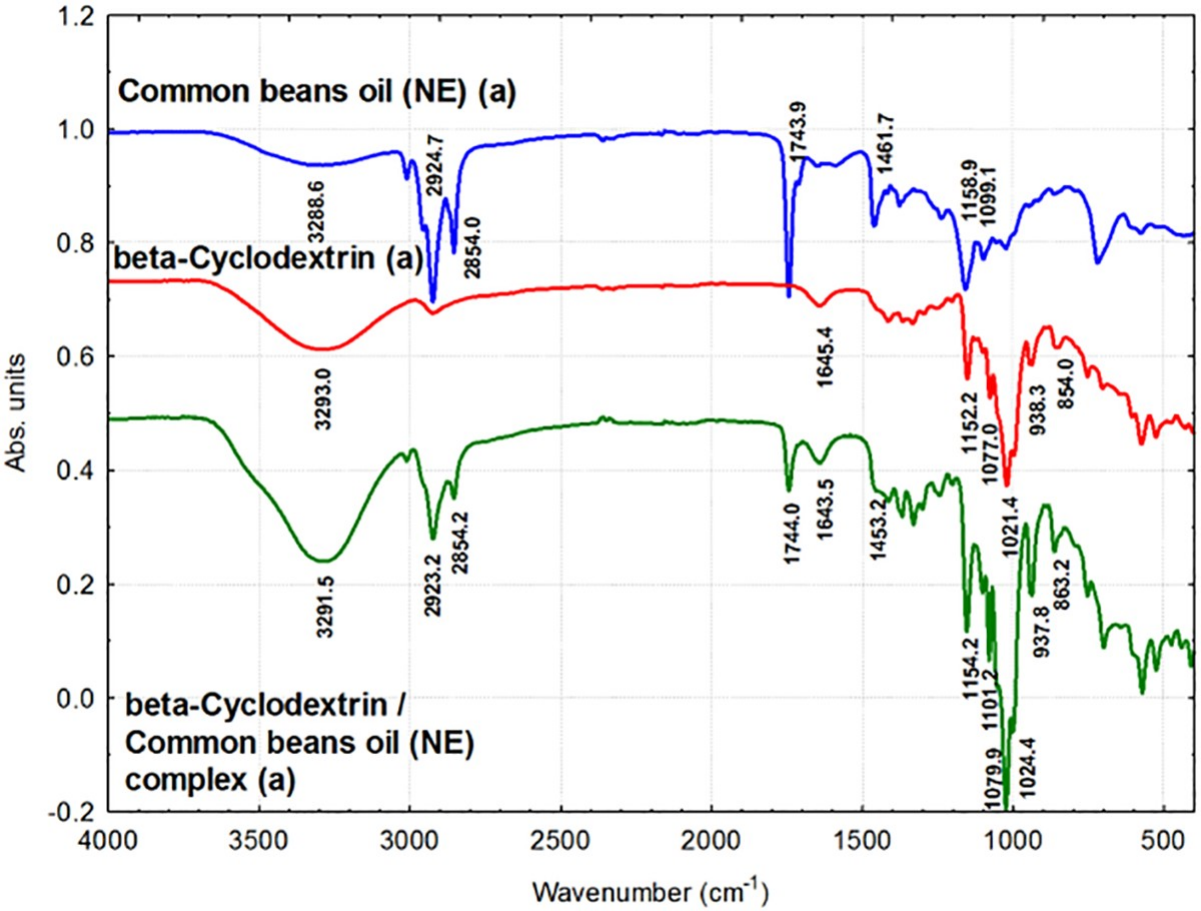
**Fourier transform infrared spectroscopy (FTIR) analysis of common beans oil (CBO-NE, blue), β-cyclodextrin (β-CD, red), and β-CD/CBO-NE complex (green).** The duplicates “a” are presented. The duplicates “b” are presented in Figure T in S1 File.

**Fig 8:**
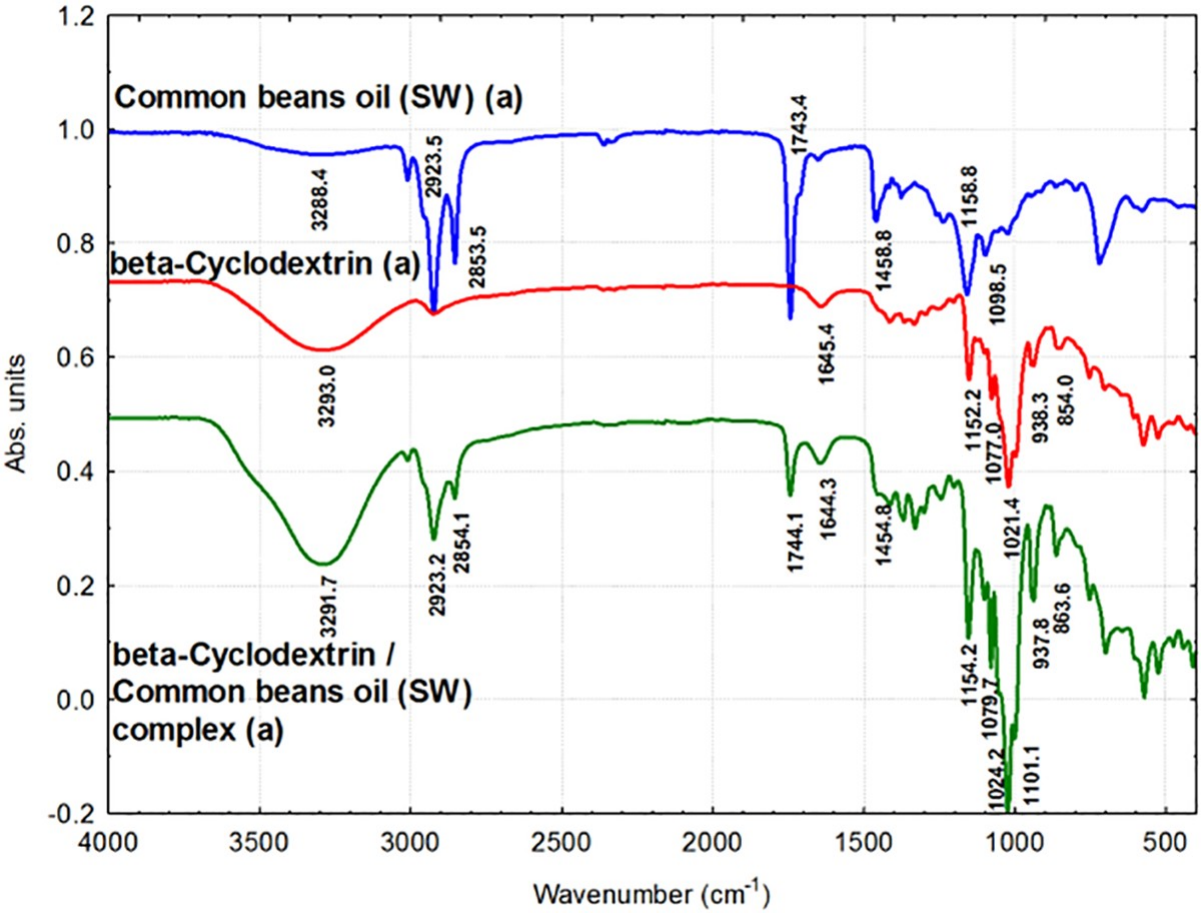
**Fourier transform infrared spectroscopy (FTIR) analysis of common beans oil (CBO-SW, blue), β-cyclodextrin (β-CD, red), and β-CD/CBO-SW complex (green).** The duplicates “a” are presented. The duplicates “b” are presented in Figure U in S1 File.

In the β-CD/CBO complexes FTIR bands that belong to both starting β-CD and CBO triglycerides can be identified. Glycerides and free FAs (FFAs) from CBO have FTIR bands corresponding to stretching vibration of O-H from water and FFAs at 3288–3289 cm^-1^, while this band appear at slightly higher wavenumber for β-CD/CBO complexes (3291.5–3291.7 cm^-1^, Figs 7 and 8, Table 3). This is due to the same band for β-CD, which appears at 3291.5–3293 cm^-1^ (Table 4). No important changes on the FTIR asymmetrical or symmetrical stretching vibration bands for C-H bonds in *cis* alkene groups or CH_2_ group appear after nanoencapsulation (3010–3010.2 cm^-1^, 2923.2 cm^-1^ for complexes and 3010.1–3010.2 cm^-1^, 2923.5–2924.7 cm^-1^ for CBO samples, Table 3). This latter band also appear in β-CD at 2924.74 (±0.94) cm^-1^ (Table 4). The similar band at 2954.2–2955.5 cm^-1^ corresponding to asymmetrical stretching vibration of C-H bond in CH_3_ group is not clearly observed in complexes, probably due to the fact that it appears as a shoulder (Figs 7 and 8). It must be noted that the FTIR band at 1743.4–1743.9 cm^-1^, corresponding to the stretching vibration of C = O bond in esters (glycerides in the case of CBO samples) also appears in the β-CD/CBO complexes at the same values (1744–1744.1 cm^-1^, Figs 7 and 8, Table 3). The band corresponding to asymmetrical bending of CH_2_ group also appears in both starting CBO samples and complexes, but at lower wavenumbers for the later (1458.8–1461.7 cm^-1^ and 1453.2–1454.8 cm^-1^, respectively, Figs 7 and 8, Table 3). The band corresponding to various O-H bending vibrations in β-CD generally appears at slightly lower values for complexes, according to Figs 7 and 8, Table 4. These bands appear for β-CD at 1645.4 (±1.04) cm^-1^, 1415.9 (±0.92) cm^-1^ and 1334.8 (±0.87) cm^-1^, in comparison with the values for β-CD/CBO complexes at 1643.5–1644.3 cm^-1^, 1414.4–1414.5 cm^-1^ and 1330.6–1330.7 cm^-1^, respectively (Table 4). A similar situation was observed for γ vibrations of CH_2_ in lipids, in comparison with the corresponding bands in β-CD/CBO complexes (1158.8–1158.9 cm^-1^ and 1154.2 cm^-1^, respectively, Table 3). However, these values can be influenced by the presence of a C-O-C glucoside stretching vibration in β-CD at 1152.2 cm^-1^ (Table 4). On the contrary, no such behavior was observed for the stretching vibration of C-O-C group in triglycerides at 1098.5–1099.1 cm^-1^ in comparison with the value of 1101.1–1101.2 cm^-1^ in the complex (Table 3). C-O and C-C stretching vibrations at 1077.0 (±0.07) cm^-1^ and 1021.4 (±0.49) cm^-1^ in β-CD appear at slightly higher values in β-CD/CBO complexes (1079.7–1079.9 cm^-1^ and 1024.2–1024.4 cm^-1^, respectively, Table 4). The same behavior was observed for δ bending vibration of C-C-H α-type glycoside bond, from 854.0 (±0.01) cm^-1^ in β-CD to 863.2–863.6 cm^-1^ in complexes (Table 4). The stretching vibration of C-H bond in β-CD ring is not significantly affected by complexation (938.3 cm^-1^ in β-CD and 937.8 cm^-1^ in complexes, Table 4). The ATR-FTIR data for the second duplicate of CBO-NE, CBO-SW, β-CD and corresponding β-CD/CBO complexes are presented in Figures T and U in S1 File.

**Table 3:** Fourier transform infrared spectroscopy (FTIR) band assignments for the main groups found in the spectrum of common bean lipid fractions from the North-East and South-West of Romania (CBO-NE and CBO-SW, respectively) and for the corresponding β-cyclodextrin complexes (β-CD/CBO-NE and β-CD/CBO-SW, respectively).

Band assignment related to common bean lipid fractions	CBO-NE Wavenumber (cm^-1^)	CBO-SW Wavenumber (cm^-1^)	β-CD/CBO-NE complex Wavenumber (cm^-1^)	β-CD/CBO-SW complex Wavenumber (cm^-1^)
ν (O-H) of water, stretching vibration	3288.55±0.09	3288.36±1.98	3291.54±0.04	3291.68±0.10
ν (C-H) of *cis* alkene groups, stretching vibration	3010.05±0.39	3010.18±0.03	3009.98±0.17	3010.18±0.12
ν_as_ (C-H) from methyl (-CH_3_) groups, asymmetrical stretch	2955.54±0.04	2954.15±1.05	-	-
ν_as_ (C-H) from methylene (CH_2_) groups, asymmetrical stretch	2924.68±0.97	2923.48±0.00	2923.17±0.10	2923.21±0.26
ν_s_ (C-H) from methylene (CH_2_) groups, symmetrical stretch	2853.96±0.21	2853.48±0.00	2854.17±0.01	2854.07±0.17
ν(C = O) of ester functional groups, stretching vibration	1743.88±0.50	1743.40±0.02	1743.98±0.20	1744.05±0.26
δ_as_(CH_2_) from methylene (CH_2_) groups, bending	1461.70±0.23	1458.78±0.08	1453.19±2.30	1454.81±0.00
γ (CH_2_) of lipids	1158.85±1.05	1158.81±0.18	1154.20±0.04	1154.16±0.00
ν_s_ (C-O-C) of triglycerides	1099.12±0.07	1098.49±0.27	1101.19±0.06	1101.08±0.03

The wavenumbers corresponding to the band assignments are presented as the mean ± standard deviation (SD).

**Table 4:** Fourier transform infrared spectroscopy (FTIR) band assignments for the main groups found in β-cyclodextrin (β-CD) and for the corresponding β-cyclodextrin / common bean oil complexes (β-CD/CBO-NE and β-CD/CBO-SW, respectively).

Band assignment related to β-cyclodextrin	β-CD Wavenumber (cm^-1^)	β-CD/CBO-NE complex Wavenumber (cm^-1^)	β-CD/CBO-SW complex Wavenumber (cm^-1^)
ν (O-H) of water, stretching vibration of O-H from β-CD	3292.98±0.00	3291.54±0.04	3291.68±0.10
ν_as_ (C-H) stretching vibration of β-CD	2924.74±0.94	2923.17±0.10	2923.21±0.26
δ (O-H) bending vibration	1645.39±1.04	1643.49±1.71	1644.33±1.72
δ (O-H) in plane bending vibration	1415.90±0.92	1414.44±0.46	1414.48±0.65
δ (O-H) bending vibration	1334.83±0.87	1330.74±0.03	1330.63±0.00
ν_s_ (C-O-C) glucosydic stretching vibration	1152.22±0.02	1154.20±0.04	1154.16±0.00
ν (C-C) stretching vibrations	1077.03±0.07	1079.87±0.03	1079.74±0.13
ν (C-O) stretching vibrations	1021.37±0.49	1024.36±0.01	1024.22±0.03
ν (C-H) stretching vibrations from cyclodextrin ring	938.26±0.34	937.78±0.00	937.77±0.02
δ (C-C-H) bending of α-type glycosidic bond	853.98±0.01	863.22±0.14	863.61±0.09

The wavenumbers corresponding to the band assignments are presented as the mean ± standard deviation (SD).

### PXRD analysis of β-CD/CBO complexes and starting compounds

**Fig 9:**
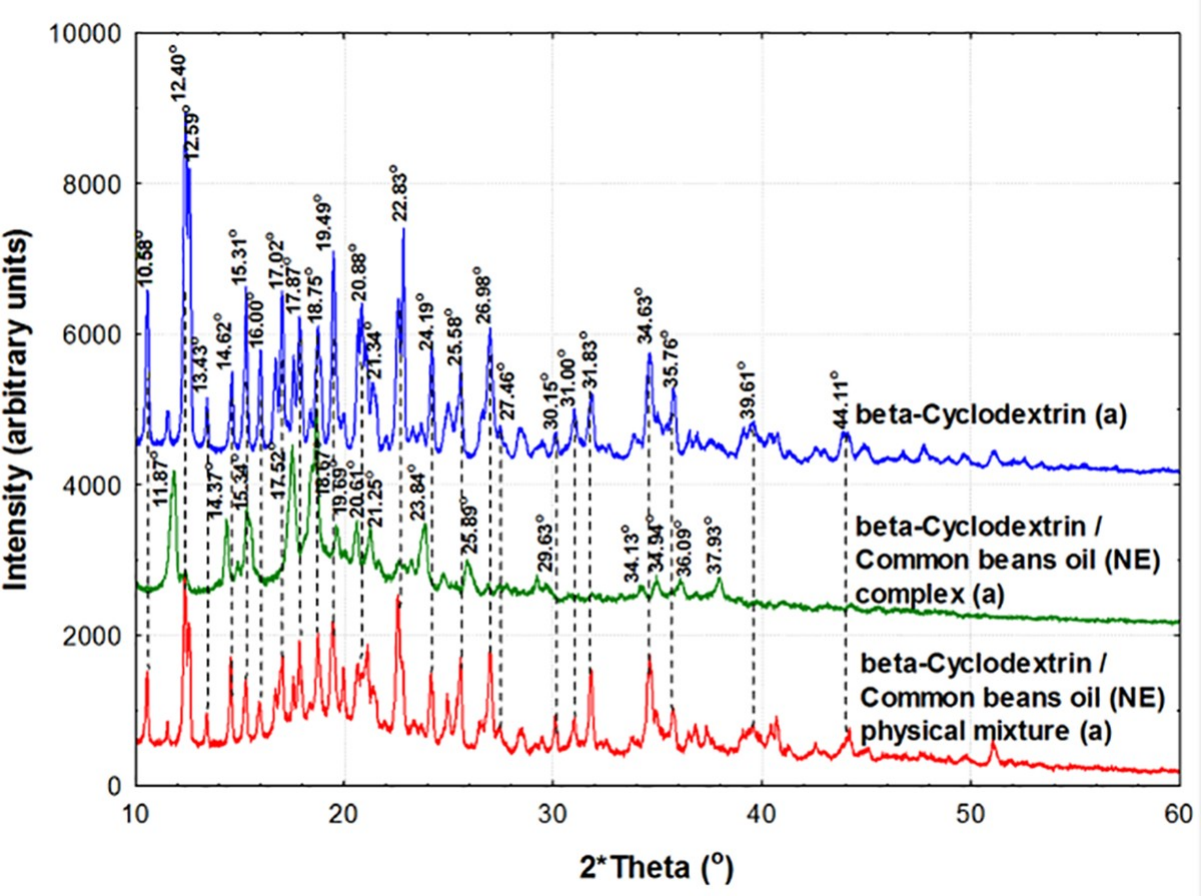
**Powder X-Ray diffractometry (PXRD) analysis of β-cyclodextrin (β-CD, blue), β-CD/CBO-NE (North-East) complex (green), and β-CD + CBO-NE physical mixture (red).** The duplicates “a” diffractograms are presented. The duplicates “b” diffractograms are presented in Figure V in S1 File.

**Fig 10:**
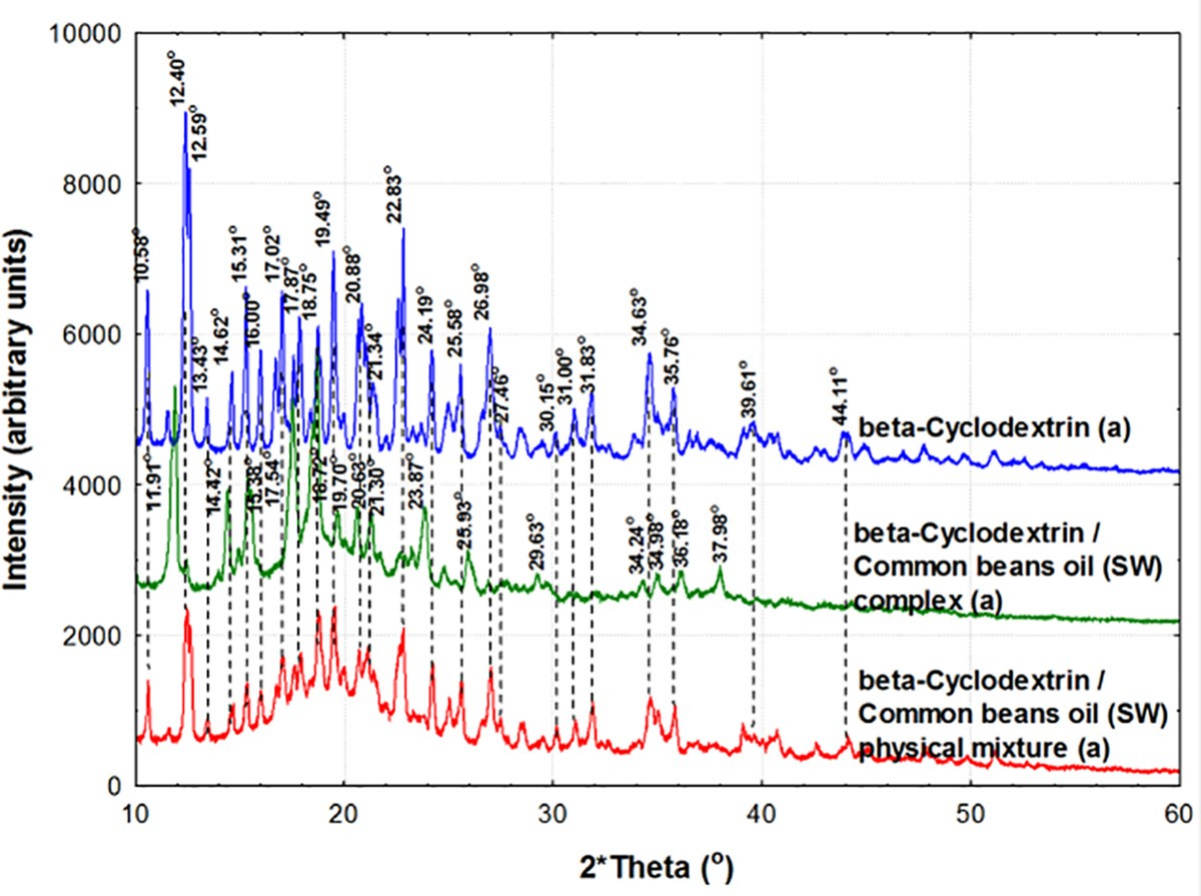
**Powder X-Ray diffractometry (PXRD) analysis of β-cyclodextrin (β-CD, blue), β-CD/CBO-SW (South-West) complex (green), and β-CD + CBO-SW physical mixture (red).** The duplicates “a” diffractograms are presented. The duplicates “b” diffractograms are presented in Figure W in S1 File.

β-CD hydrate has a well formed crystalline structure that provide a clear PXRD diffractogram. The main specific 2θ signals appear at 10.57 (±0.02), 12.37 (±0.04), 12.60 (±0.02), 13.42 (±0.02)), 14.60 (±0.04), 15.29 (±0.02), 15.98 (±0.02), 17.00 (±0.03), 17.86 (±0.02), 18.72 (±0.05), 19.47 (±0.03), 20.85 (±0.02), 21.35 (±0.00), 22.80 (±0.04), 24.18 (±0.02), 25.57 (±0.01), 26.97 (±0.01), 27.44 (±0.03), 30.13 (±0.03), 30.99 (±0.01), 31.83 (±0.00), 34.62 (±0.02), 35.76 (±0.01), 39.60 (±0.02) and 44.10 (±0.01)° for β-CD hydrate (Figs 9 and 10). The same 2θ signals also appear in the physical mixtures of β-CD and CBO samples, but more attenuated. It is especially the case of 10.59 (±0.03), 12.43 (±0.06), 12.58 (±0.04), 13.44 (±0.05), 14.62 (±0.05), 15.32 (±0.04), 15.98 (±0.04), 17.03 (±0.02), 17.89 (±0.03), 18.77 (±0.02), 19.47 (±0.04), 20.81 (±0.04), 22.78 (±0.03), 24.18 (±0.03), 25.58 (±0.04), 27.00 (±0.02), 27.45 (±0.03), 30.14 (±0.04), 31.04 (±0.03), 31.84 (±0.06), 34.64 (±0.02), 35.78 (±0.05), 39.60 (±0.02), and 44.13 (±0.04)° for β-CD mixed with CBO-NE or CBO-SW in a physical mixture (mean values for all duplicates “a” and “b”, see Figs 9 and 10, Figures V and W in S1 File for duplicates “b”. Significantly different results on PXRD analysis were obtained for β-CD/CBO complexes. These 2θ signals were especially identified in the following regions for β-CD/CBO complexes (average of four samples, corresponding to duplicates “a” and “b” of β-CD/CBO-NE and β-CD/CBO-SW complexes, see Figs 9 and 10, Figures V and W in S1 File): 11.89 (±0.02), 14.40 (±0.03), 15.36 (±0.02), 17.53 (±0.02), 18.69 (±0.02), 19.68 (±0.02), 20.62 (±0.02), 21.28 (±0.02), 23.86 (±0.02), 25.91 (±0.03), 29.67 (±0.05), 34.95 (±0.03) and 36.11 (±0.05)°.

The formation of the host-guest inclusion complexes drastically influence the crystal structure of the final product, as was observed by PXRD analysis of β-CD/CBO complexes in comparison with β-CD hydrate and its physical mixture with CBO samples. Moreover, a higher degree of disorder appear into the crystals during the nanoencapsulation process. This can be quantified by calculating the crystallinity index, expressed as the percent ratio of the intensity of the crystalline peak and total diffracted intensity, which was former used to evaluate the crystallinity of carbohydrates [37]. The crystallinity index considerably decreases from 93.28 (±5.31) % for β-CD hydrate to 60.02 (±22.08) % and 60.85 (±20.28) % for β-CD/CBO-NE and β-CD/CBO-SW complexes, respectively. The crystallinity index for physical mixtures is also low due to the presence of the common beans lipid fractions that considerably attenuate the diffractions (values of 69.05 (±19.97) % and 66.99 (±23.86) % for NE and SW cases, respectively).

### Encapsulation efficiency, fatty acid profile, and controlled release of the encapsulated CBO from the β-CD/CBO complexes

Encapsulation efficiency (EE) is calculated as the percent ratio between the encapsulated CBO and the total oil from the complex (the sum of surface and encapsulated oil fractions). However, it is difficult to evaluate exactly the fraction of included glycerides inside the cavity of cyclodextrins (providing encapsulated oil) and the fraction of completely non-encapsulated glycerides (which can be located at the surface of crystals and between cyclodextrin entities; this fraction is considered surface oil). This is due to the fact that triglycerides have three hydrophobic fatty acid moieties that are compatible with CD cavities. The glycerol moiety remain outside of the cavity, being more hydrophilic. As a consequence, the surface oil can be separated by short extraction with appropriate solvent (hexane), while encapsulated oil requires multiple extractions for a longer time. In the case of β-CD/CBO complexes, no oil can be separated at the fourth hexane extraction of the residual complex. Thus, the surface oil was separated by short extraction with hexane (one minute), while the encapsulated oil was separated by three subsequent hexane extractions. The calculated EE were higher in the case β-CD/CBO-SW complexes and little bit lower for β-CD/CBO-NE samples. However, the standard deviations are relatively high, especially due to the non-homogenous materials having above-mentioned interactions between constituents (60 (±18.46) % and 42.26 (±4.17) %, respectively).

The FA profile of encapsulated oil (containing triglycerides as the major constituents) is different from the raw CBO samples. This is due to the different hydrophobic (van der Waals) interactions of saturated and unsaturated fatty acid moieties with the β-CD cavities. As a result, the total relative concentration of SFAs slightly increased after β-CD nanoencapsulation (to 22.35–24.7%, Table 1), while the total relative concentrations of MUFAs and especially PUFAs increased, the last from 58.82 (±1.14) % to 43.36 (±7.31) % for β-CD/CBO-NE samples and from 55.74 (±0.06) % to 35.13 (±3.78) % for β-CD/CBO-NE complexes. It can be seen that the standard deviations are considerably higher for encapsulated oils due to the above-mentioned non-homogeneity of complexes. The most important increase of the relative concentration was observed for palmitic acid (from 13.90 (±1.80) % to 17.41 (±3.74) % for β-CD/CBO-NE complexes and from 17.36 (±0.41) % to 18.71 (±6.64) % for β-CD/CBO-SW complexes, Table 1). On the other hand, linoleic acid (the most important PUFA) considerably decreases from 43.4 (±1.95) % to 28.96 (±6.28) % for β-CD/CBO-NE and from 35.23 (±0.68) % to 20.34 (±5.19) % for β-CD/CBO-SW cases (Table 1). The most valuable omega-3 FA, namely α-linolenic acid (as methyl ester) had similar relative concentrations in both raw and encapsulated CBOs (values of 13.13 (±0.59) % and 15.72 (±0.30) % for the raw oils, 14.04 (±1.54) % and 12.41 (±1.95) % for the encapsulated oils, respectively; Table 1). As a result, the omega-3/omega-6 ratio significantly increased after β-CD nanoencapsulation of CBOs. These values are approximately two-thirds higher in oils included into the β-CD cavities (0.51 (±0.07) for encapsulated CBO-NE oil and only 0.32 (±0.02) for the raw sample; 0.76 (±0.26) for CBO-SW oil in the complex, in comparison with the 0.51 (±0.01) in the raw sample; Table 1). However, all values are higher than the minimum value of 0.2 that was considered to provide valuable omega-3 based oils, having healthy effects for the cardio-vascular and neuronal disorders. All GC chromatograms of the encapsulated CBO-NE and CBO-SW oils are presented in Figures A-D in S1 File.

**Fig 11:**
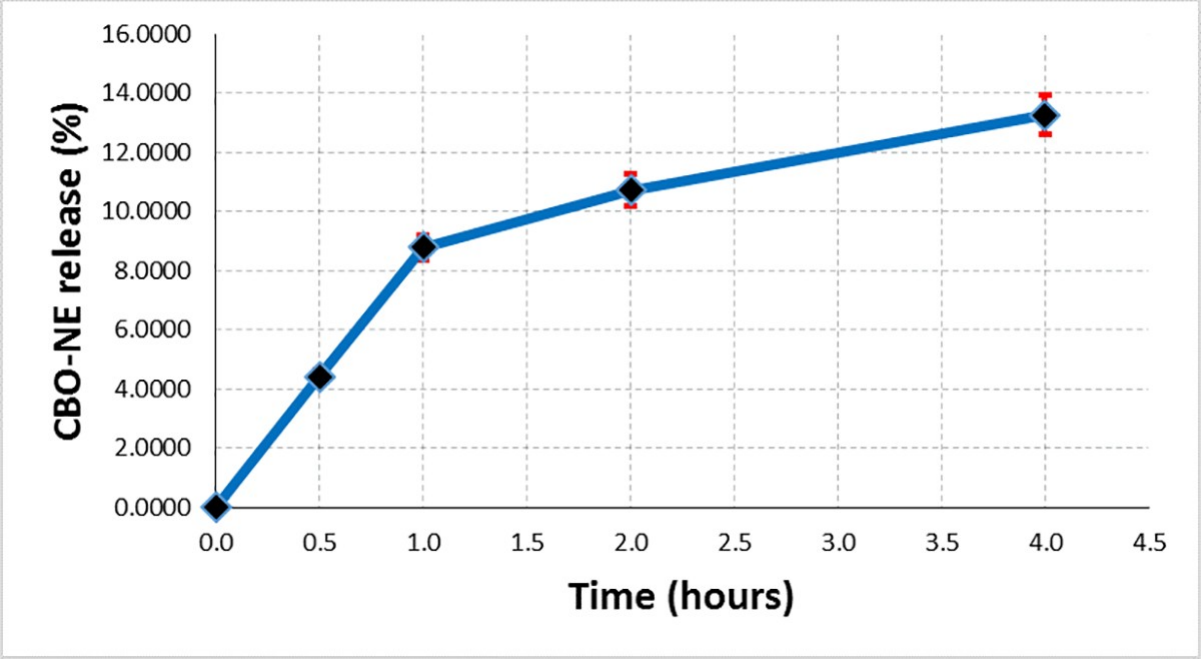
Controlled release of CBO-NE encapsulated oil (%) from its β-CD complex.

**Fig 12:**
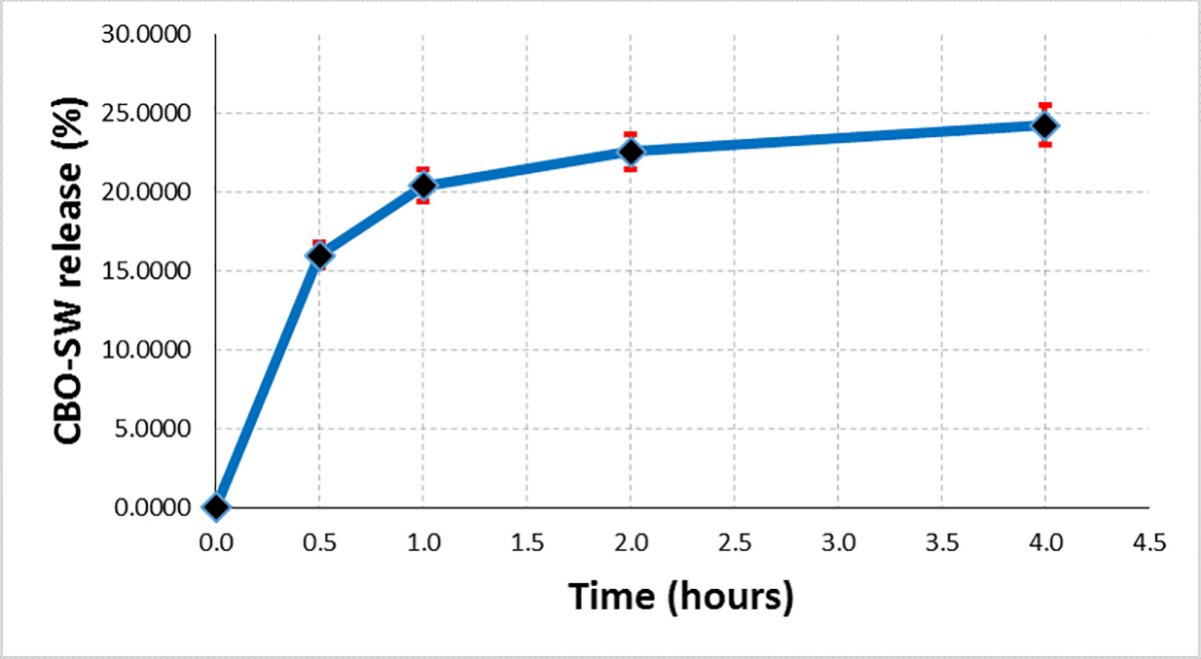
Controlled release of CBO-SW encapsulated oil (%) from its β-CD complex.

The controlled release of the very hydrophobic guest compounds (mainly, a mixture of triglycerides) from the β-CD/CBO complexes can only be performed if a second hydrophobic layer is considered together with the classical aqueous one. Due to the fact that FA glycerides (especially omega-3 ones) can be released even in a food matrix and/or biological systems (both having continuous aqueous layer and possible discontinuously hydrophobic parts–e.g., oily fractions in food products and cell membrane in biological systems), a sodium chloride solution of 0.9% concentration have been used. This NaCl solution simulated the aqueous layer, while hexane simulated the hydrophobic part in food and biological systems [26]. The controlled release of CBO from β-CD/CBO complexes was performed at room temperature (~25°C) under vigorously stirring (at a speed of magnetic stirrer of 750 rpm) for four hours. Exact volumes of hexane solution were separated from the upper layer time to time and subjected to evaporation in the dark at room temperature until no mass change was observed. The cumulative masses of separated oil were converted to percent of the CBO released, knowing the total oil encapsulated from the EE data. The CBO release (%) was plotted against time. In the Figs 11 and 12 are presented the controlled release results as the average values of duplicate analyses (including percent standard error bars). It was observed that both β-CD/CBO-NE and β-CD/CBO-SW complexes present logarithmic behavior for controlled release of the encapsulated oil. However, this release continues after four hours of analysis (the mean value of the released oil fraction was 13.3% for β-CD/CBO-NE complex and 24.2% for β-CD/CBO-SW complex; Figs 11 and 12). This behavior also suggest possible controlled release of this omega-3 contained products (common beans lipid fractions or other beans ingredients) from cyclodextrin complexes used in food supplements and/or functional food products.

## Conclusion

In this study the stabilization of the common beans lipid fractions from the North-East and South-West of Romania by β-cyclodextrin complexation have been for the first time evaluated. The FA profile of the CBO samples reveals a high content of omega-3 FAs (as glycerides), with α-linolenic acid as the main compound. This valuable but less stable FA was identified in the CBO samples at a concentration of 13.1–15.7%, providing an omega-3/omega-6 ratio of 0.31–0.52. This ratio generates healthy properties for these oily materials. The β-CD complexation of these lipid fractions were performed by kneading with recovering yields over 70%. The host-guest molecular interactions between β-CD cavity and glycerides contained by CBO samples take place in complex and competitive ways. The formation of the molecular inclusion compounds were evaluated by thermal methods, as well as by ATR-FTIR and PXRD. TG-DTG and DSC analyses reveal the decrease of the hydration water content in β-CD/CBO complexes and the high relative content of “surface” water molecules in complexes. On the other hand, the DSC crystalline/amorphous transition peak completely disappears after nanoencapsulation. The formation of the amorphous β-CD/CBO complex is also supported by PXRD data. The crystallinity index consistently decreases from 93.3% for β-CD hydrate to 60–60.9% for complexes. All measurements confirm the formation of inclusion complexes between β-CD and triglycerides from common beans lipid fractions. Consequently, β-CD can be used for stabilizing (and possible controlled release) of fatty acid glycerides, especially omega-3 ones (with increased omega-3/omega-6 ratio at almost double values), from Romanian common beans (*Phaseolus vulgaris* L.) based ingredients that can be used in various food supplements or even functional food products.

## Supporting information

S1 File GC-MS analysis of the derivatized CBO samples, FTIR, PXRD, TG-DTG and DSC analyses of CBO, β-CD and β-CD/CBO complexes. Gas chromatograms from the GC-MS analysis of the derivatized lipid fraction of beans (CBO-NE, duplicate “a”–Figure A, CBO-NE, duplicate “b”–Figure B, CBO-SW, duplicate “a”–Figure C, CBO-SW, duplicate “b”–Figure D; raw–top, and encapsulated—bottom). MS spectra of myristic acid–Figure E, petroselinic acid–Figure F, pentadecanoic acid–Figure G, palmitoleic acid–Figure H, palmitic acid–Figure I, margaric acid–Figure J, linoleic acid–Figure K, α-linolenic acid–Figure L, oleic acid–Figure M, elaidic acid–Figure N, stearic acid–Figure O, arachidic acid–Figure P, behenic acid–Figure Q, tricosanoic acid–Figure R, and lignoceric acid–Figure S (as methyl esters), from the GC-MS analysis of the derivatized beans lipid fractions (experimental–top and from the NIST database–bottom). Fourier transform infrared spectroscopy (FTIR) data for CBO-NE (blue), β-CD (red) and β-CD/CBO-NE complex (green) (duplicates “b”)–Figure T, CBO-SW (blue), β-CD (red) and β-CD/CBO-SW complex (green) (duplicates “b”)–Figure U. Powder X-Ray diffractometry (PXRD) data for β-CD (blue), β-CD/CBO-NE complex (green), β-CD + CBO-NE physical mixture (red) (duplicates “b”)–Figure V, β-CD (blue), β-CD/CBO-SW complex (green), β-CD + CBO-SW physical mixture (red) (duplicates “b”)–Figure W. Thermogravimetry-differential thermogravimetry (TG-DTG) analysis of β-cyclodextrin (β-CD, duplicate “b”)–Figure X, β-cyclodextrin / common beans (North-East) oil complex (β-CD / CBO-NE, duplicate “b”)–Figure Y, β-cyclodextrin / common beans (South-West) oil complex (β-CD / CBO-SW, duplicate “b”)–Figure Z. Differential scanning calorimetry (DSC) analysis of β-cyclodextrin (β-CD, duplicate “b”)–Figure AA, β-cyclodextrin / common beans (North-East) oil complex (β-CD / CBO-NE, duplicate “b”)–Figure AB, β-cyclodextrin / common beans (South-West) oil complex (β-CD / CBO-SW, duplicate “b”)–Figure AC.
(PDF)
