# Supplementary material for: Fatty acid profile of Romanian’s common bean (Phaseolus vulgaris L.) lipid fractions and their complexation ability by β-cyclodextrin
Source: PLoS One. 2019 Nov 22;14(11):e0225474. doi: 10.1371/journal.pone.0225474 (PMC6874339; doi:10.1371/journal.pone.0225474)
Supplement: S1 File — Gas chromatograms from the GC-MS analysis of the derivatized lipid fraction of beans (CBO-NE, duplicate “a”–Figure A, CBO-NE, duplicate “b”–Figure B, CBO-SW, duplicate “a”–Figure C, CBO-SW, duplicate “b”–Figure D; raw–top, and encapsulated—bottom). MS spectra of myristic acid–Figure E, petroselinic acid–Figure F, pentadecanoic acid–Figure G, palmitoleic acid–Figure H, palmitic acid–Figure I, margaric acid–Figure J, linoleic acid–Figure K, α-linolenic acid–Figure L, oleic acid–Figure M, elaidic acid–Figure N, stearic acid–Figure O, arachidic acid–Figure P, behenic acid–Figure Q, tricosanoic acid–Figure R, and lignoceric acid–Figure S (as methyl esters), from the GC-MS analysis of the derivatized beans lipid fractions (experimental–top and from the NIST database–bottom). Fourier transform infrared spectroscopy (FTIR) data for CBO-NE (blue), β-CD (red) and β-CD/CBO-NE complex (green) (duplicates “b”)–Figure T, CBO-SW (blue), β-CD (red) and β-CD/CBO-SW complex (green) (duplicates “b”)–Figure U. Powder X-Ray diffractometry (PXRD) data for β-CD (blue), β-CD/CBO-NE complex (green), β-CD + CBO-NE physical mixture (red) (duplicates “b”)–Figure V, β-CD (blue), β-CD/CBO-SW complex (green), β-CD + CBO-SW physical mixture (red) (duplicates “b”)–Figure W. Thermogravimetry-differential thermogravimetry (TG-DTG) analysis of β-cyclodextrin (β-CD, duplicate “b”)–Figure X, β-cyclodextrin / common beans (North-East) oil complex (β-CD / CBO-NE, duplicate “b”)–Figure Y, β-cyclodextrin / common beans (South-West) oil complex (β-CD / CBO-SW, duplicate “b”)–Figure Z. Differential scanning calorimetry (DSC) analysis of β-cyclodextrin (β-CD, duplicate “b”)–Figure AA, β-cyclodextrin / common beans (North-East) oil complex (β-CD / CBO-NE, duplicate “b”)–Figure AB, β-cyclodextrin / common beans (South-West) oil complex (β-CD / CBO-SW, duplicate “b”)–Figure AC. (PDF) [file pone.0225474.s001.pdf]

SUPPORTING INFORMATION

(*PLoS ONE*)

Fatty acid profile of Romanian's common bean (*Phaseolus vulgaris* L.) lipid fractions and their complexation ability by β-cyclodextrin

Ioan David[1¶], Manuela D. Orboi[2¶], Marius D. Simandi[1], Cosmina A. Chirilă[1], Corina I. Megyesi[1], Laura Rădulescu[1], Lavinia P. Drăghia[3], Alexandra T. Lukinich-Gruia[3], Cornelia Muntean[4,5], Daniel I. Hădărugă[6], Nicoleta G. Hădărugă[1*]

[1] Department of Food Science, Banat's University of Agricultural Sciences and Veterinary Medicine "King Michael I of Romania" from Timişoara, Timişoara, Romania
[2] Department of Economics and Company Financing, Banat's University of Agricultural Sciences and Veterinary Medicine "King Michael I of Romania" from Timişoara, Timişoara, Romania
[3] Centre for Gene and Cellular Therapies in the Treatment of Cancer – OncoGen, Clinical County Hospital of Timişoara, Timişoara, Romania
[4] Department of Applied Chemistry and Engineering of Inorganic Compounds and Environment, Polytechnic University of Timişoara, Timişoara, Romania
[5] Research Institute for Renewable Energy, Polytechnic University of Timişoara, Timişoara, Romania
[6] Department of Applied Chemistry, Organic and Natural Compounds Engineering, Polytechnic University of Timişoara, Timişoara, Romania

* Corresponding author
E-mail: nico_hadaruga@yahoo.com (NGH)

¶ These authors contributed equally to this work. They are both principal authors.

Gas chromatography – mass spectrometry (GC-MS) data for the derivatized common bean lipid fractions

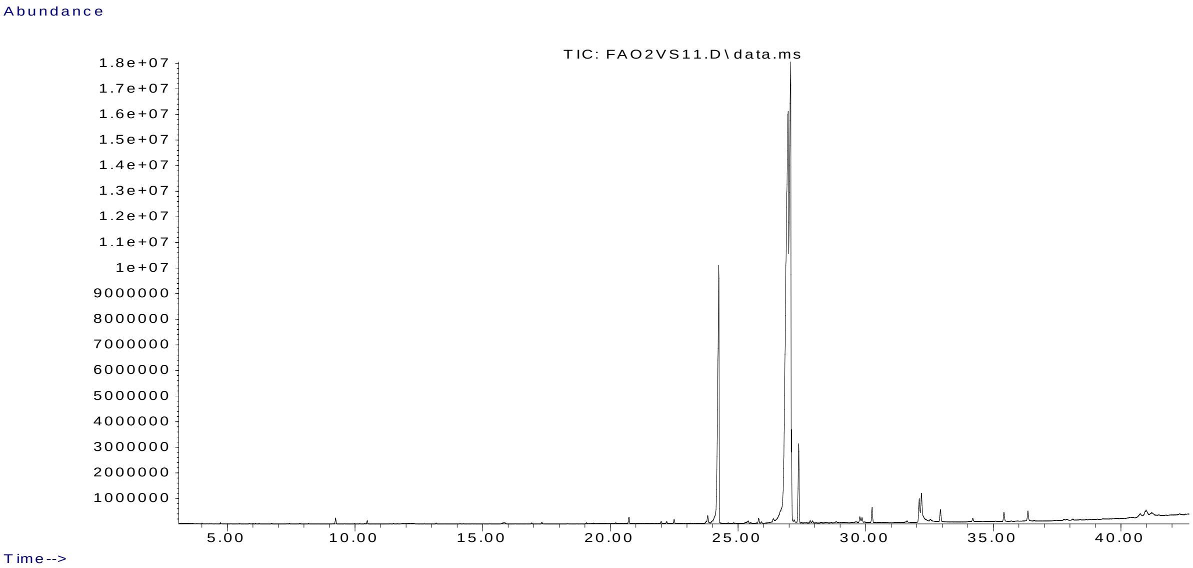

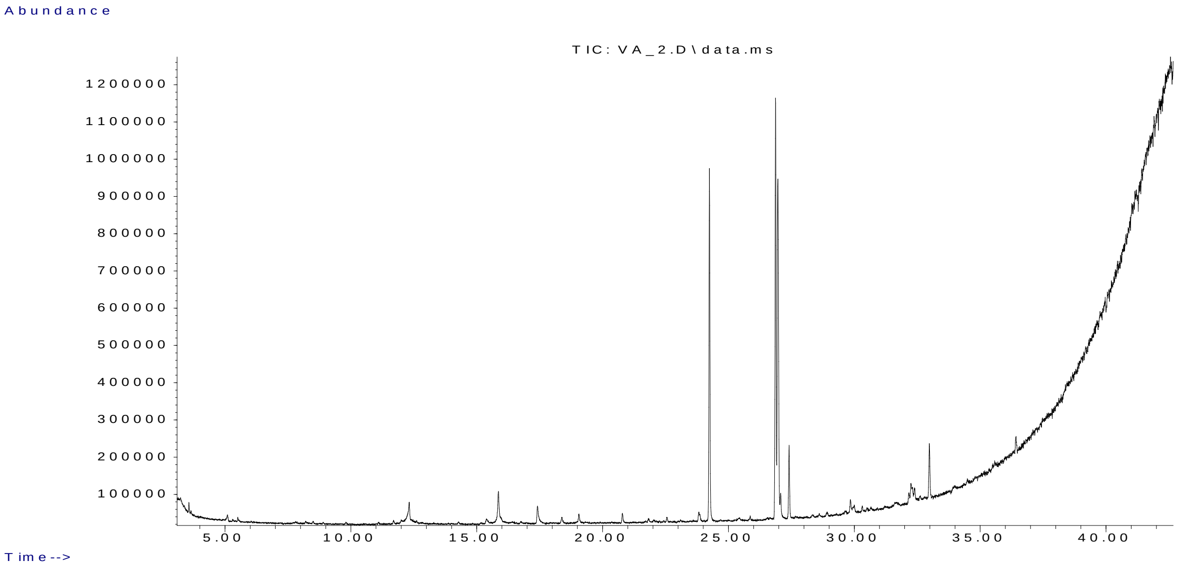

Figure A. Gas chromatogram from the GC-MS analysis of the derivatized lipid fraction of beans (code CBO-NE, duplicate "a"; raw – top, and encapsulated - bottom)

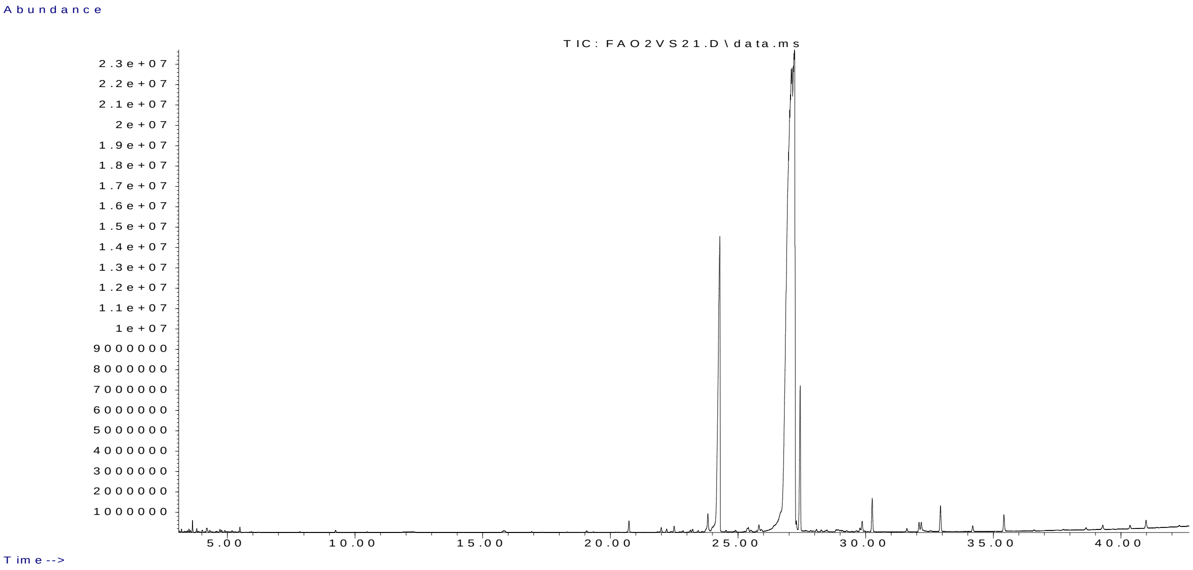

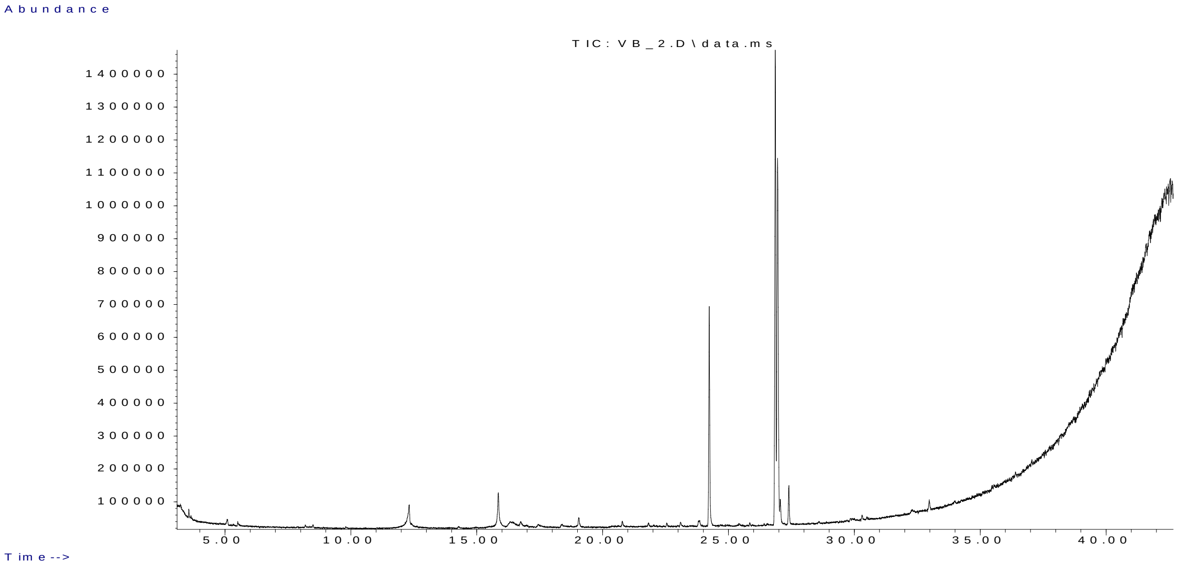

Figure B. Gas chromatogram from the GC-MS analysis of the derivatized lipid fraction of beans (code CBO-NE, duplicate "b"; raw – top, and encapsulated - bottom)

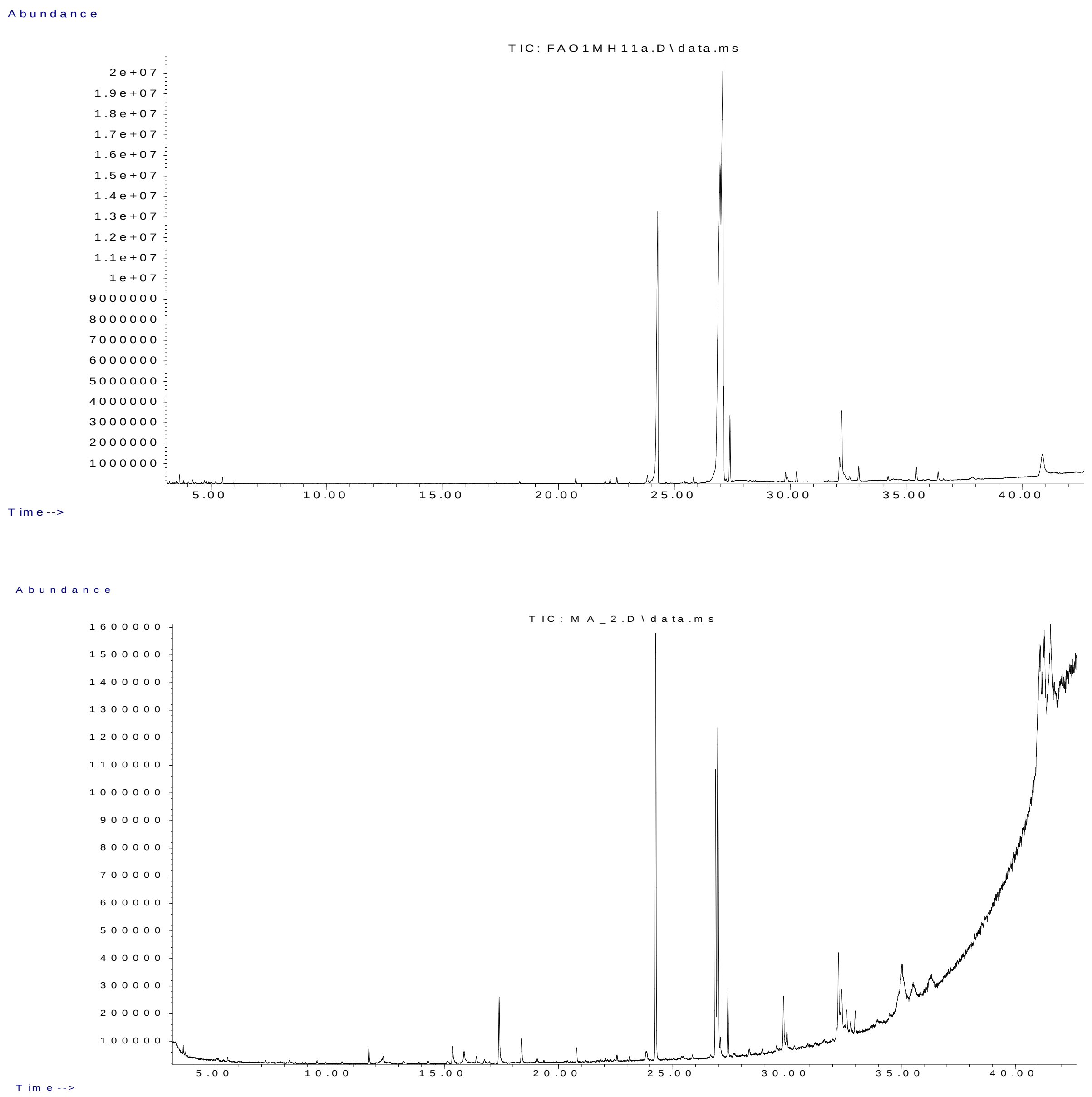

Figure C. Gas chromatogram from the GC-MS analysis of the derivatized lipid fraction of beans (code CBO-SW, duplicate "a"; raw – top, and encapsulated - bottom)

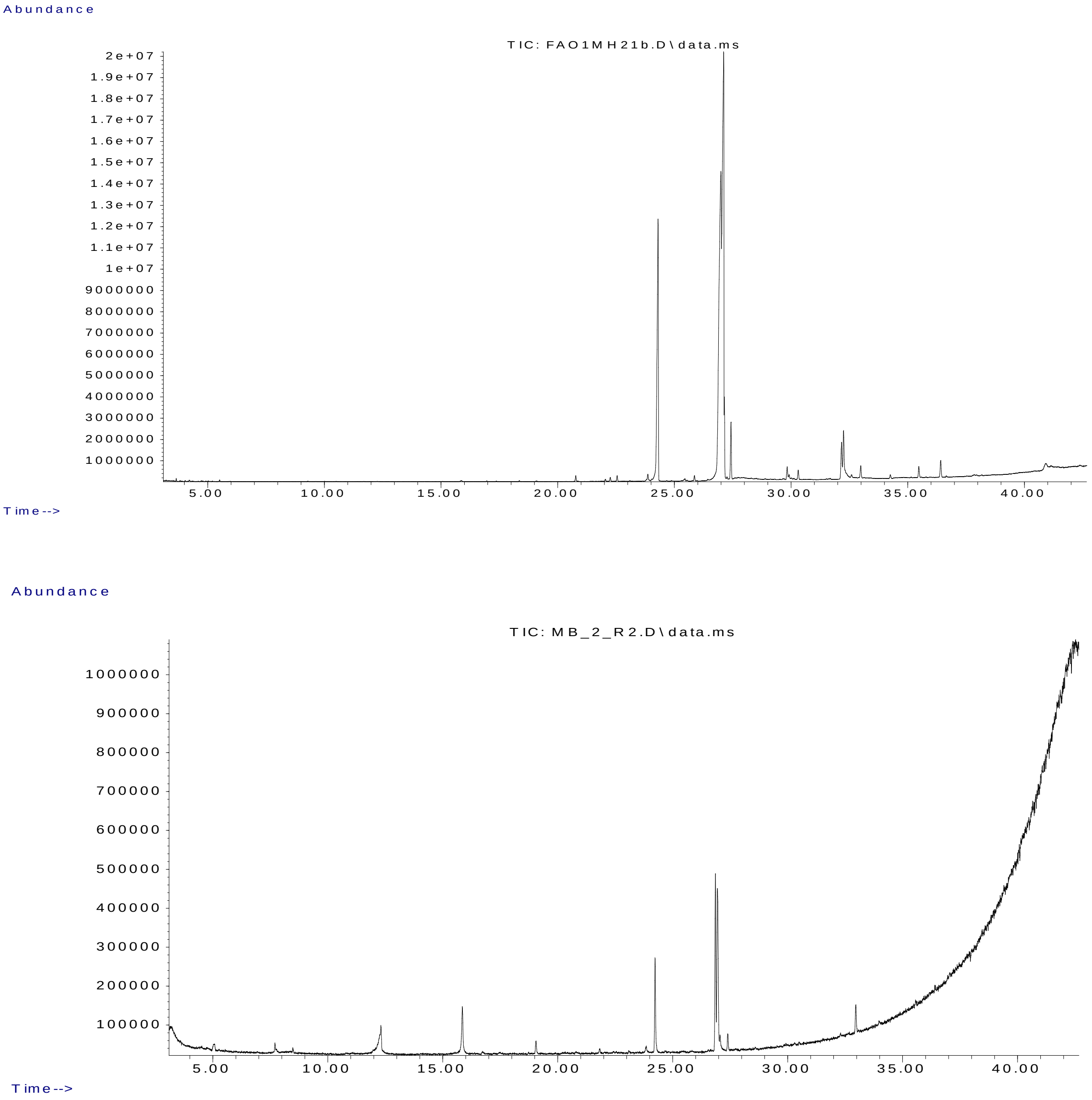

Figure D. Gas chromatogram from the GC-MS analysis of the derivatized lipid fraction of beans (code CBO-SW, duplicate “b”; raw – top, and encapsulated - bottom)

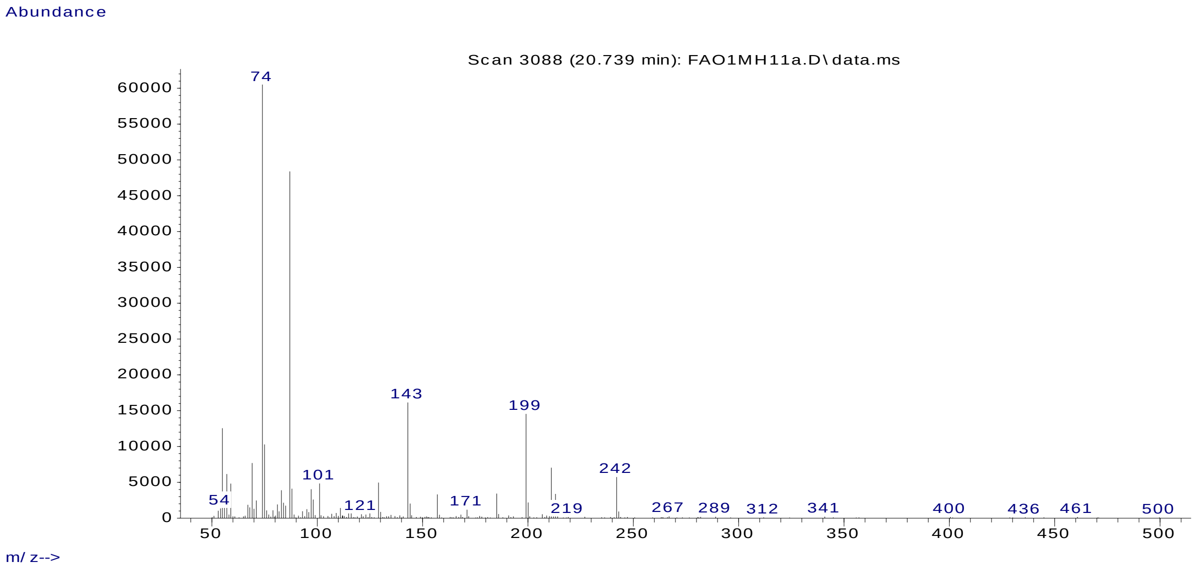

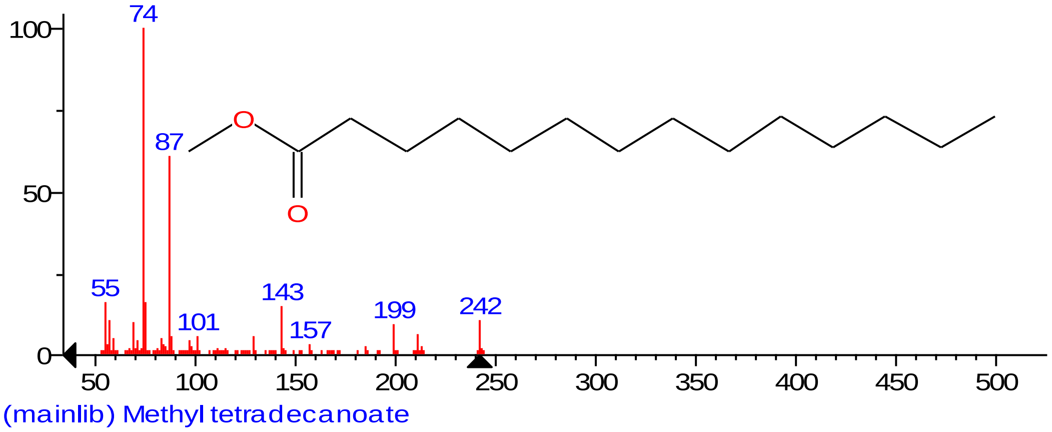

Figure E. MS spectra of myristic acid (methyl ester) from the GC-MS analysis of the derivatized beans lipid fractions (experimental – top and from the NIST database – bottom)

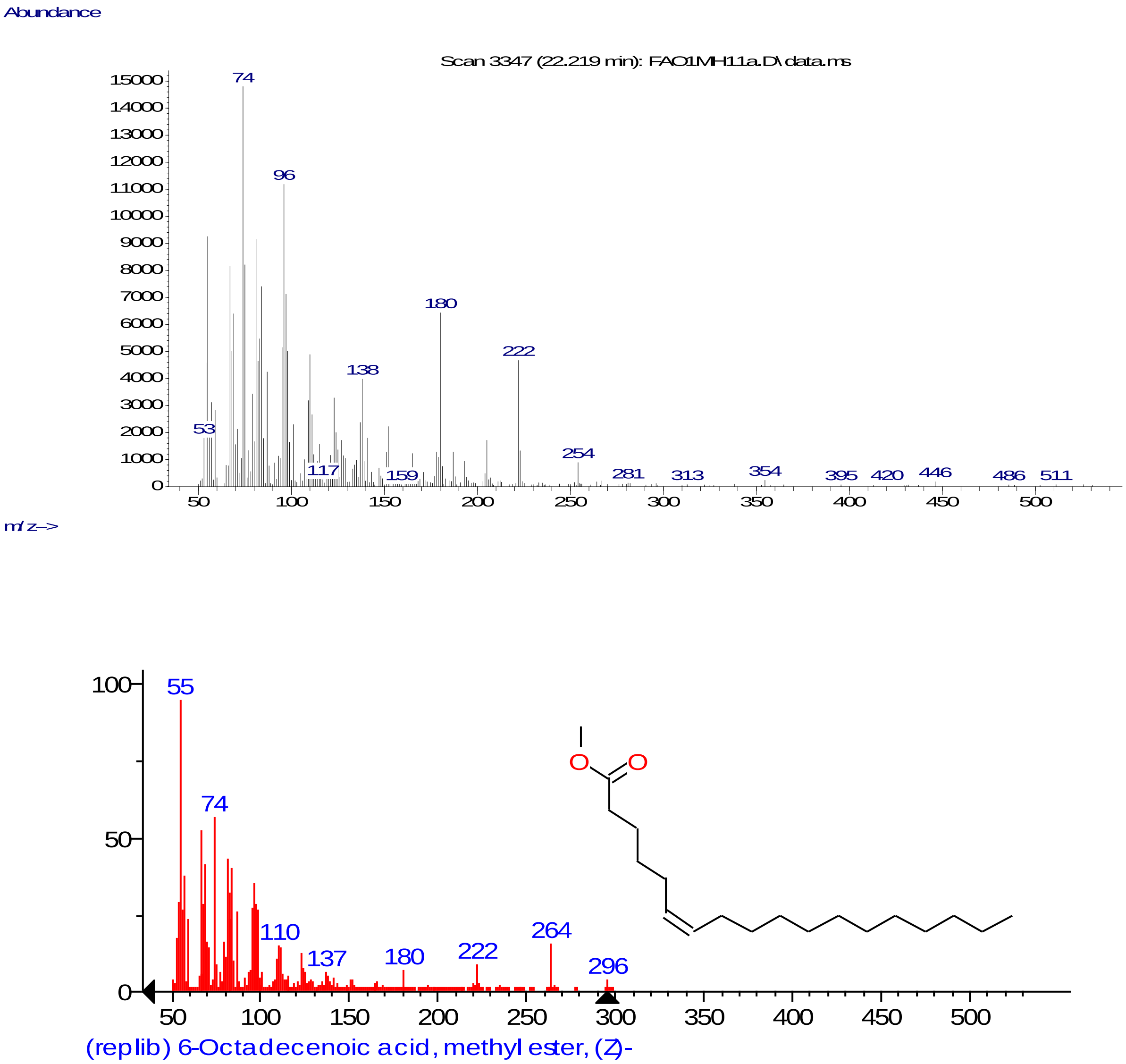

Figure F. MS spectra of petroselinic acid (methyl ester) from the GC-MS analysis of the derivatized beans lipid fractions (experimental – top and from the NIST database – bottom)

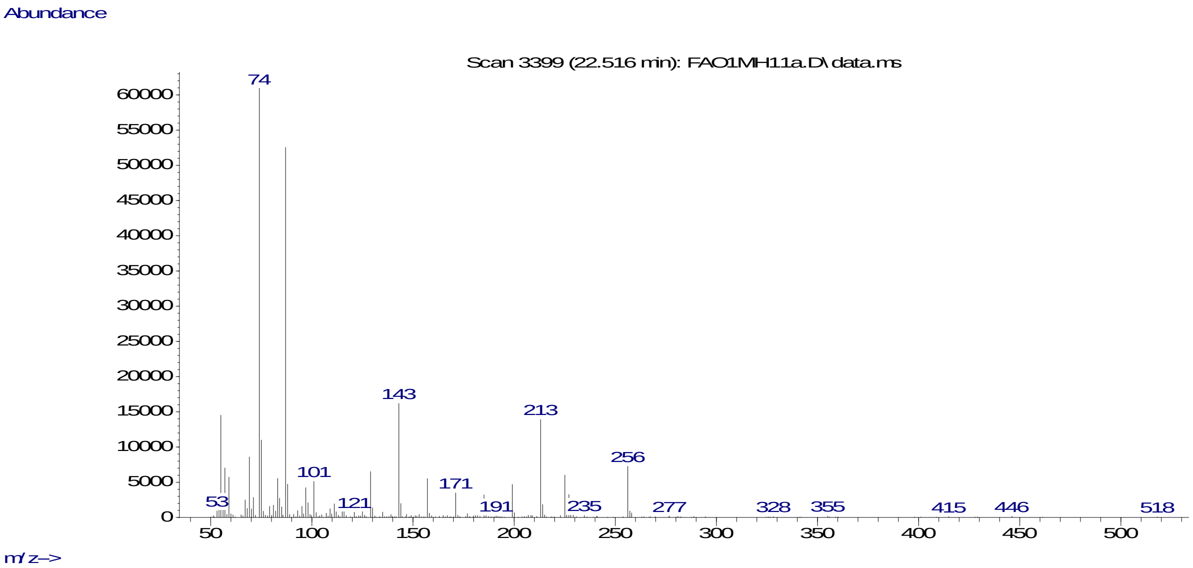

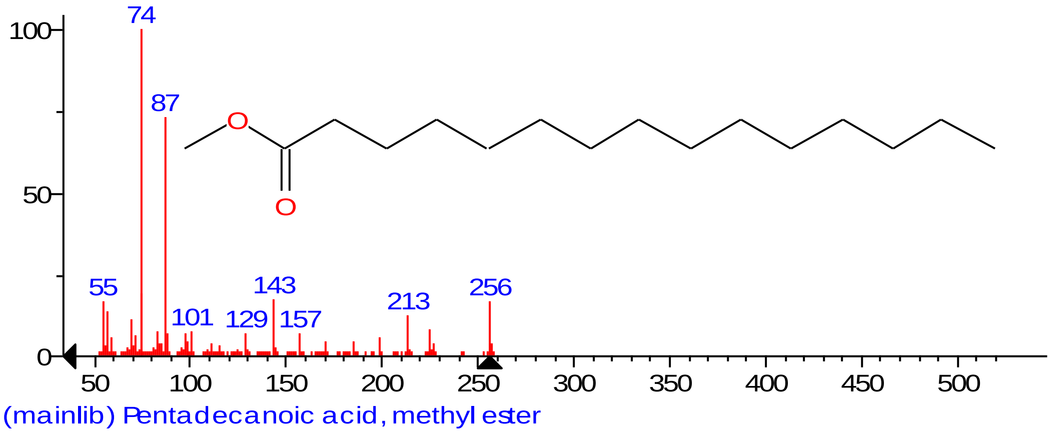

Figure G. MS spectra of pentadecanoic acid (methyl ester) from the GC-MS analysis of the derivatized beans lipid fractions (experimental – top and from the NIST database – bottom)

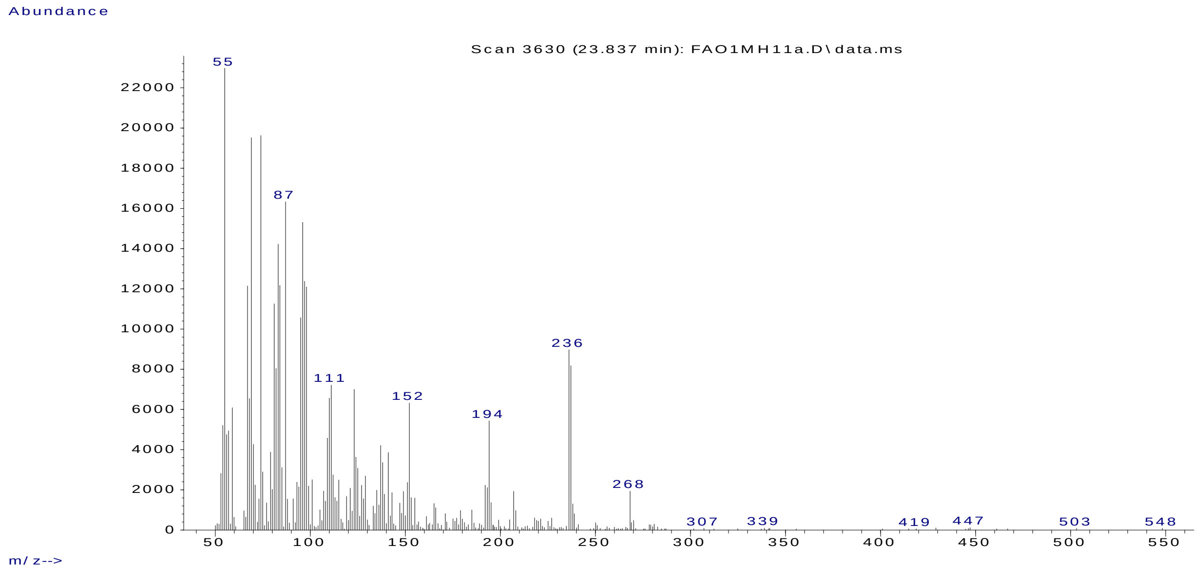

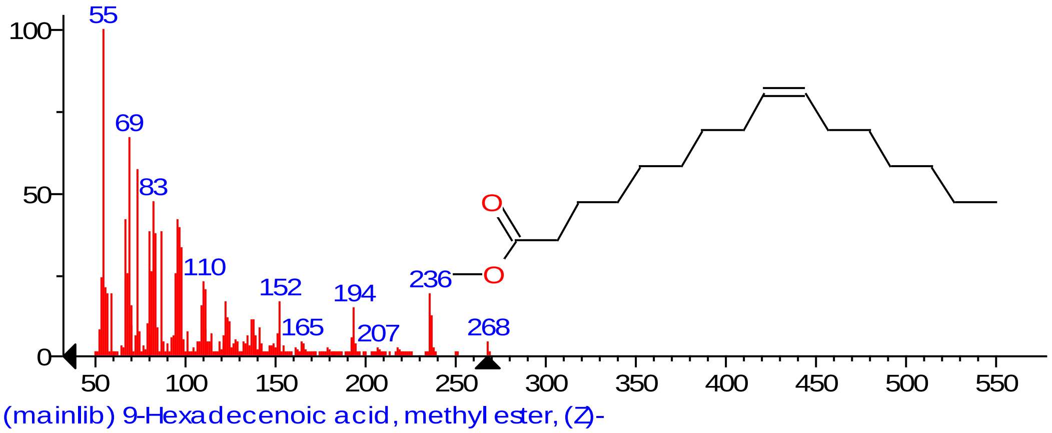

Figure H. MS spectra of palmitoleic acid (methyl ester) from the GC-MS analysis of the derivatized beans lipid fractions (experimental – top and from the NIST database – bottom)

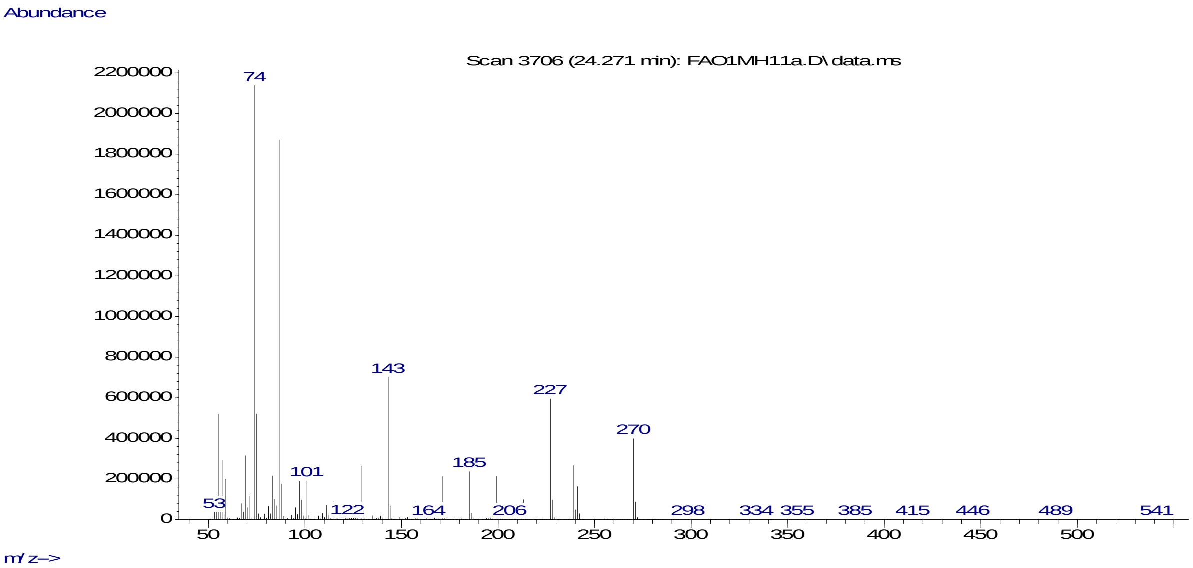

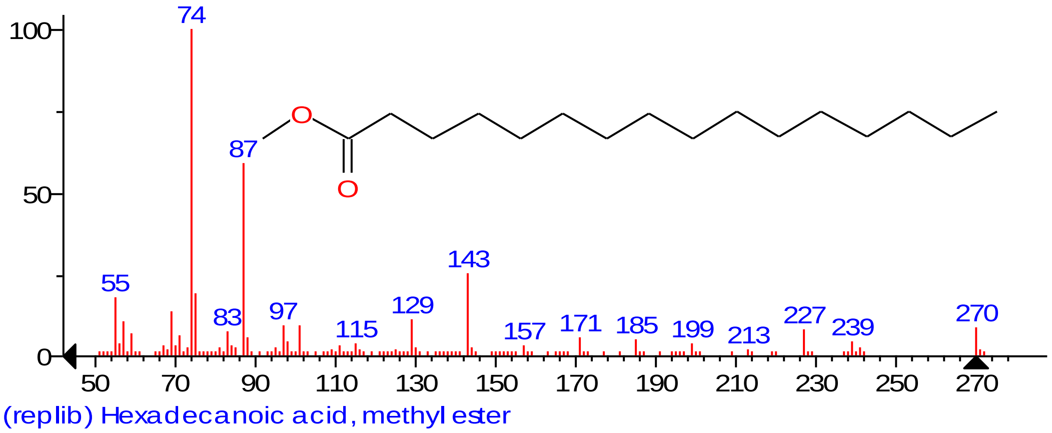

Figure I. MS spectra of palmitic acid (methyl ester) from the GC-MS analysis of the derivatized beans lipid fractions (experimental – top and from the NIST database – bottom)

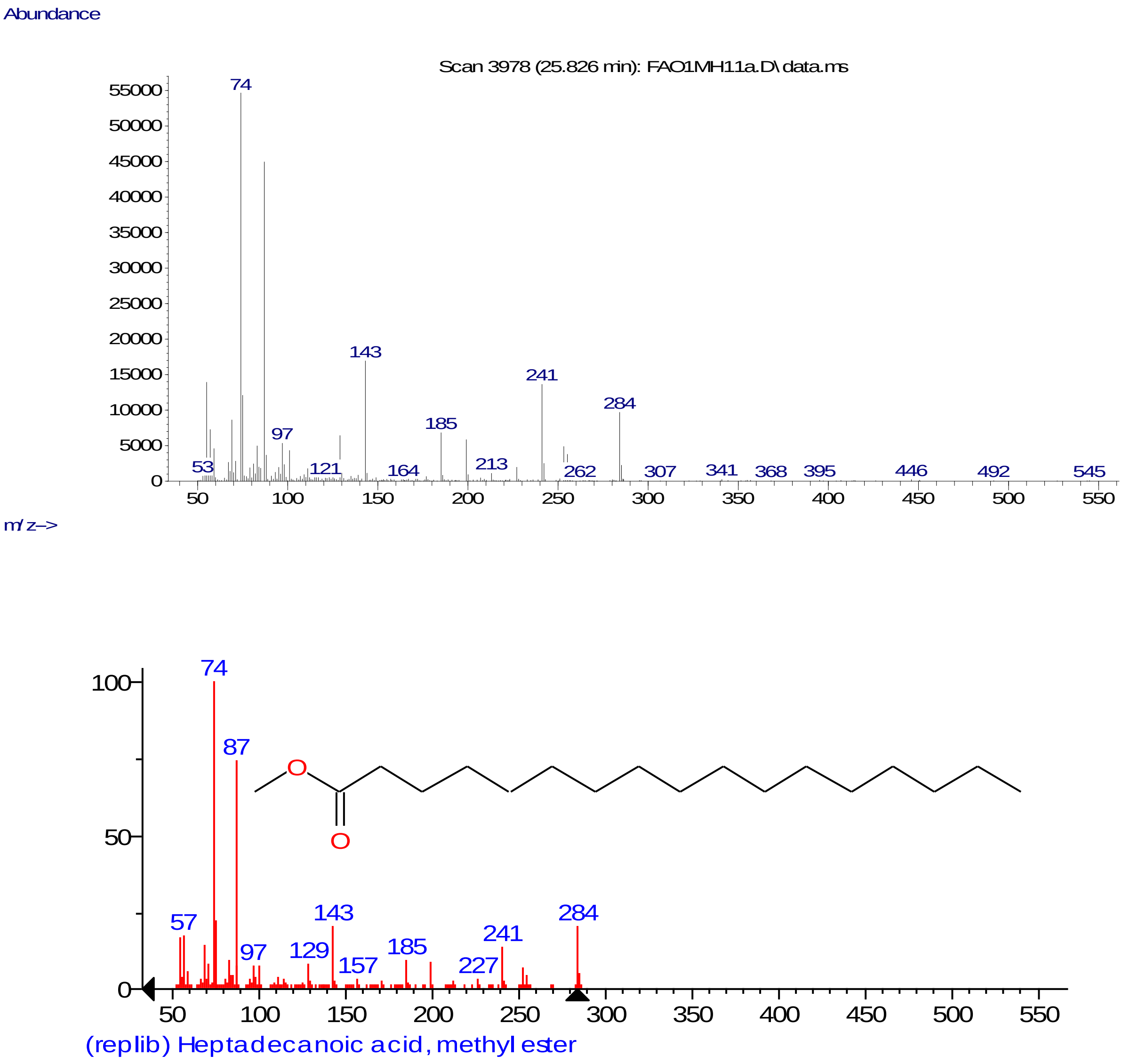

Figure J. MS spectra of margaric acid (methyl ester) from the GC-MS analysis of the derivatized beans lipid fractions (experimental – top and from the NIST database – bottom)

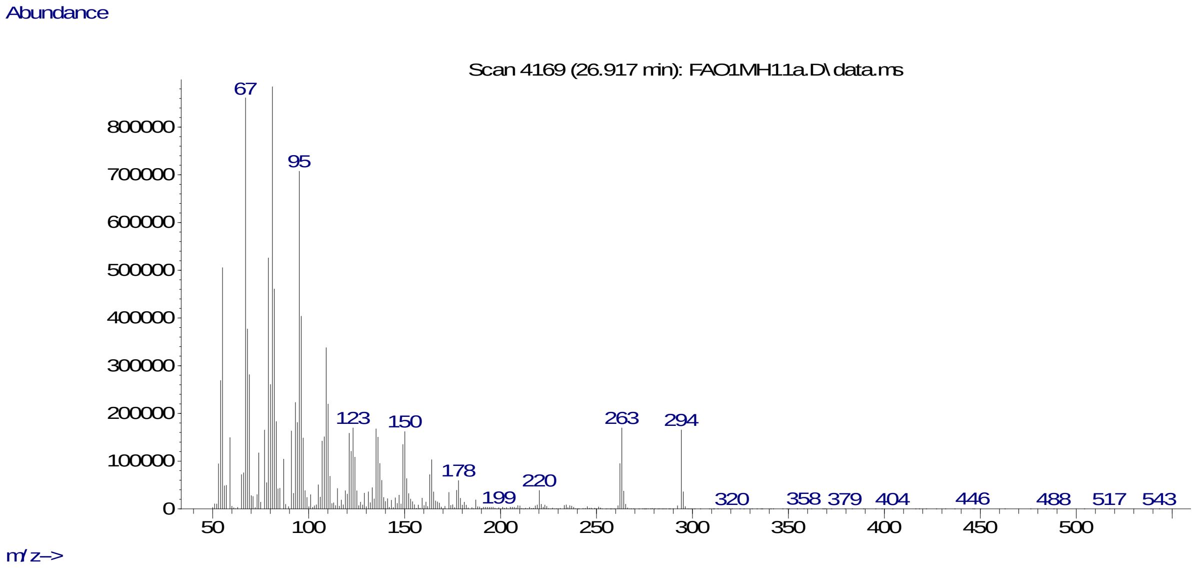

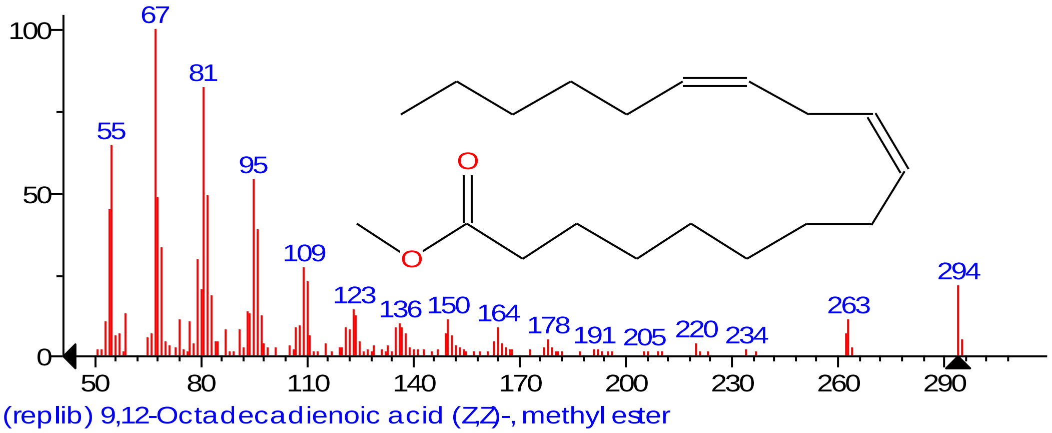

Figure K. MS spectra of linoleic acid (methyl ester) from the GC-MS analysis of the derivatized beans lipid fractions (experimental – top and from the NIST database – bottom)

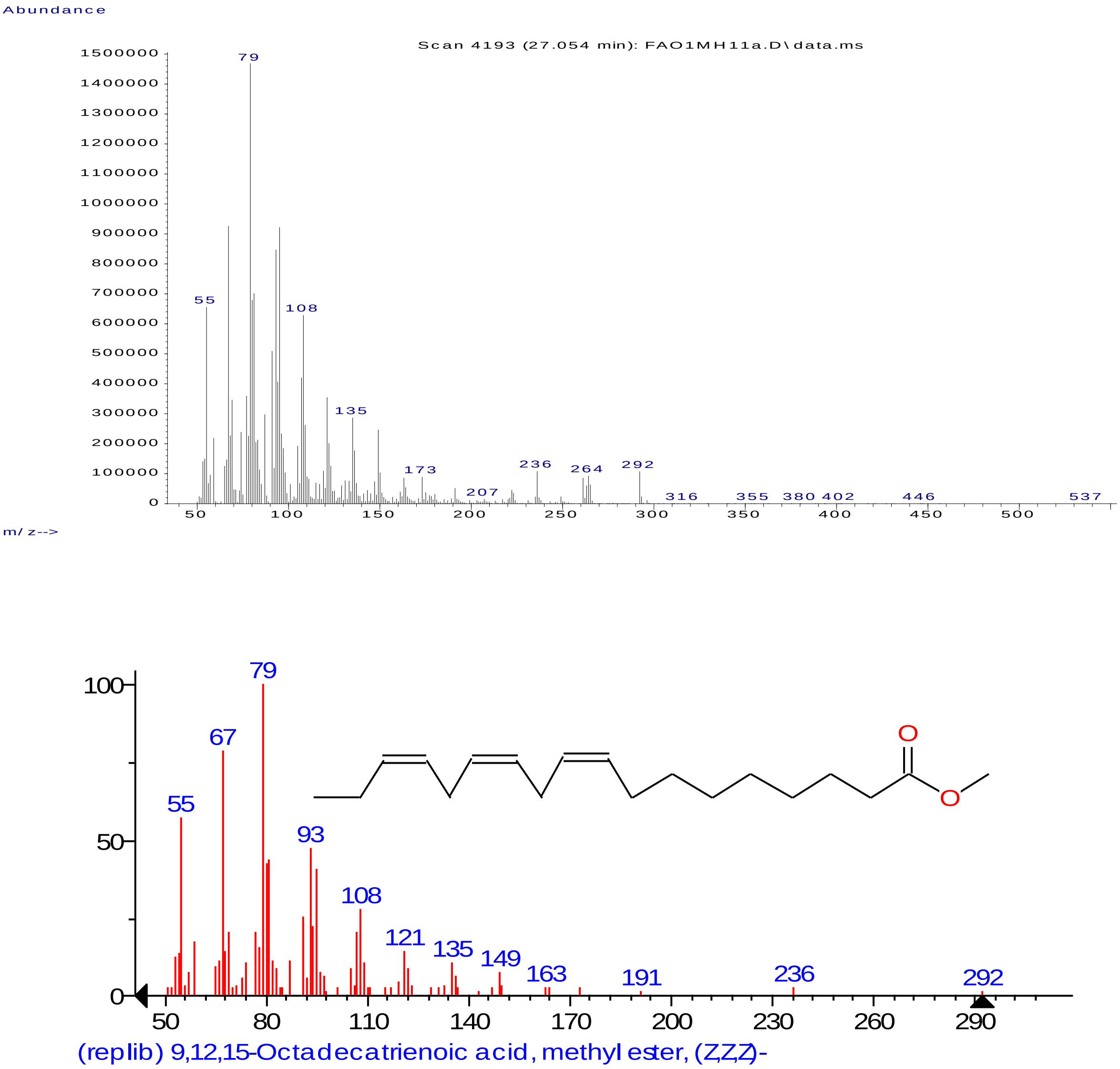

Figure L. MS spectra of α-linolenic acid (methyl ester) from the GC-MS analysis of the derivatized beans lipid fractions (experimental – top and from the NIST database – bottom)

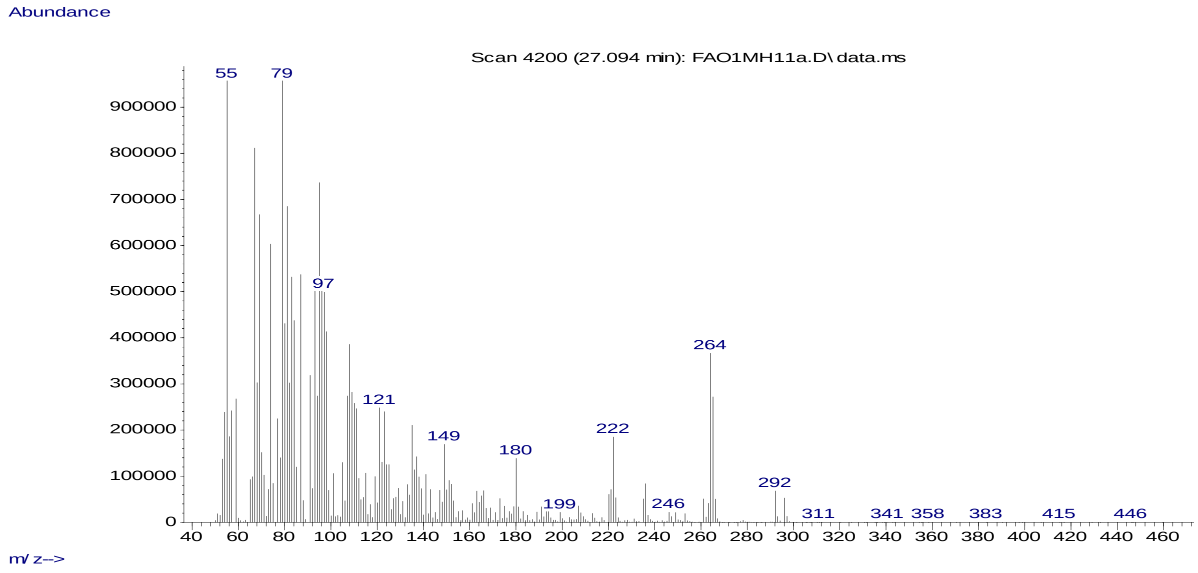

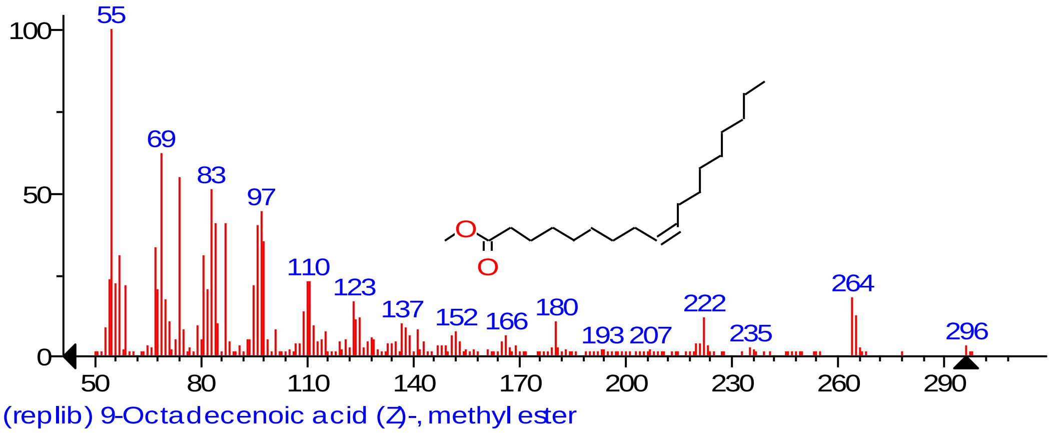

Figure M. MS spectra of oleic acid (methyl ester) from the GC-MS analysis of the derivatized beans lipid fractions (experimental – top and from the NIST database – bottom)

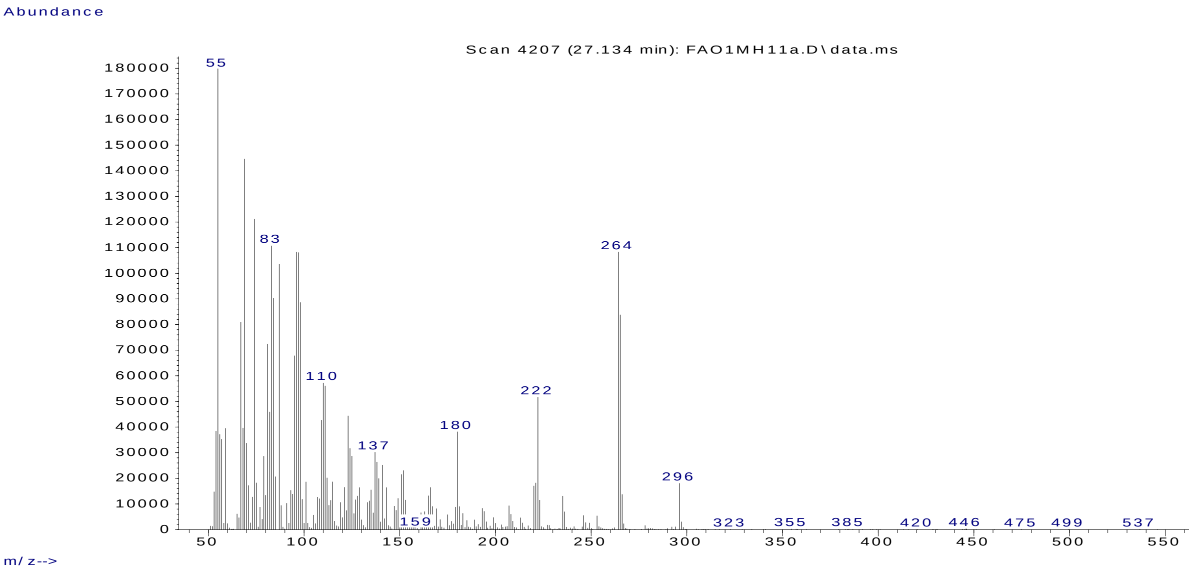

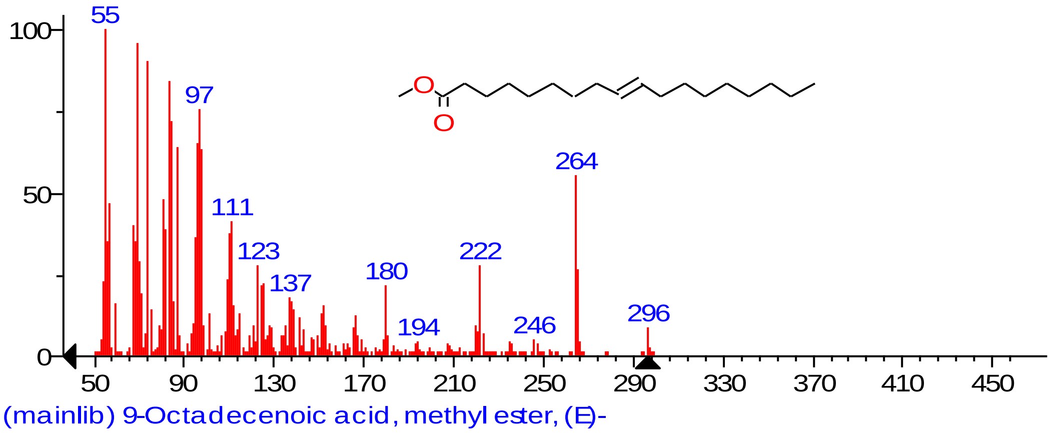

Figure N. MS spectra of elaidic acid (methyl ester) from the GC-MS analysis of the derivatized beans lipid fractions (experimental – top and from the NIST database – bottom)

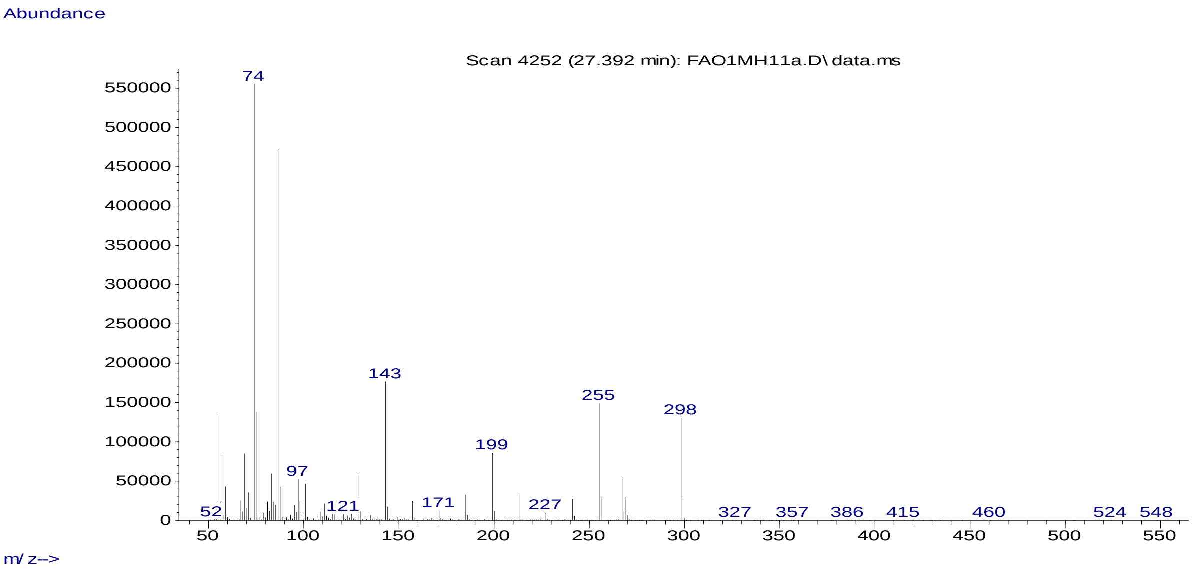

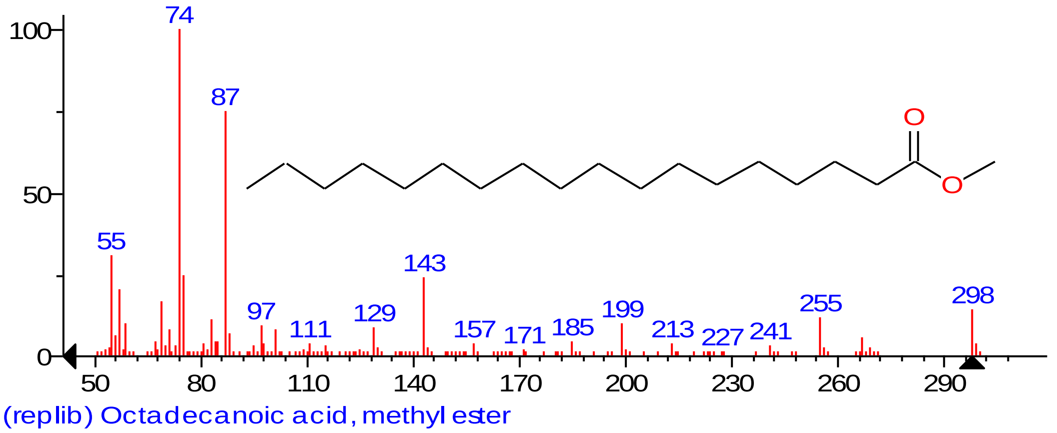

Figure O. MS spectra of stearic acid (methyl ester) from the GC-MS analysis of the derivatized beans lipid fractions (experimental – top and from the NIST database – bottom)

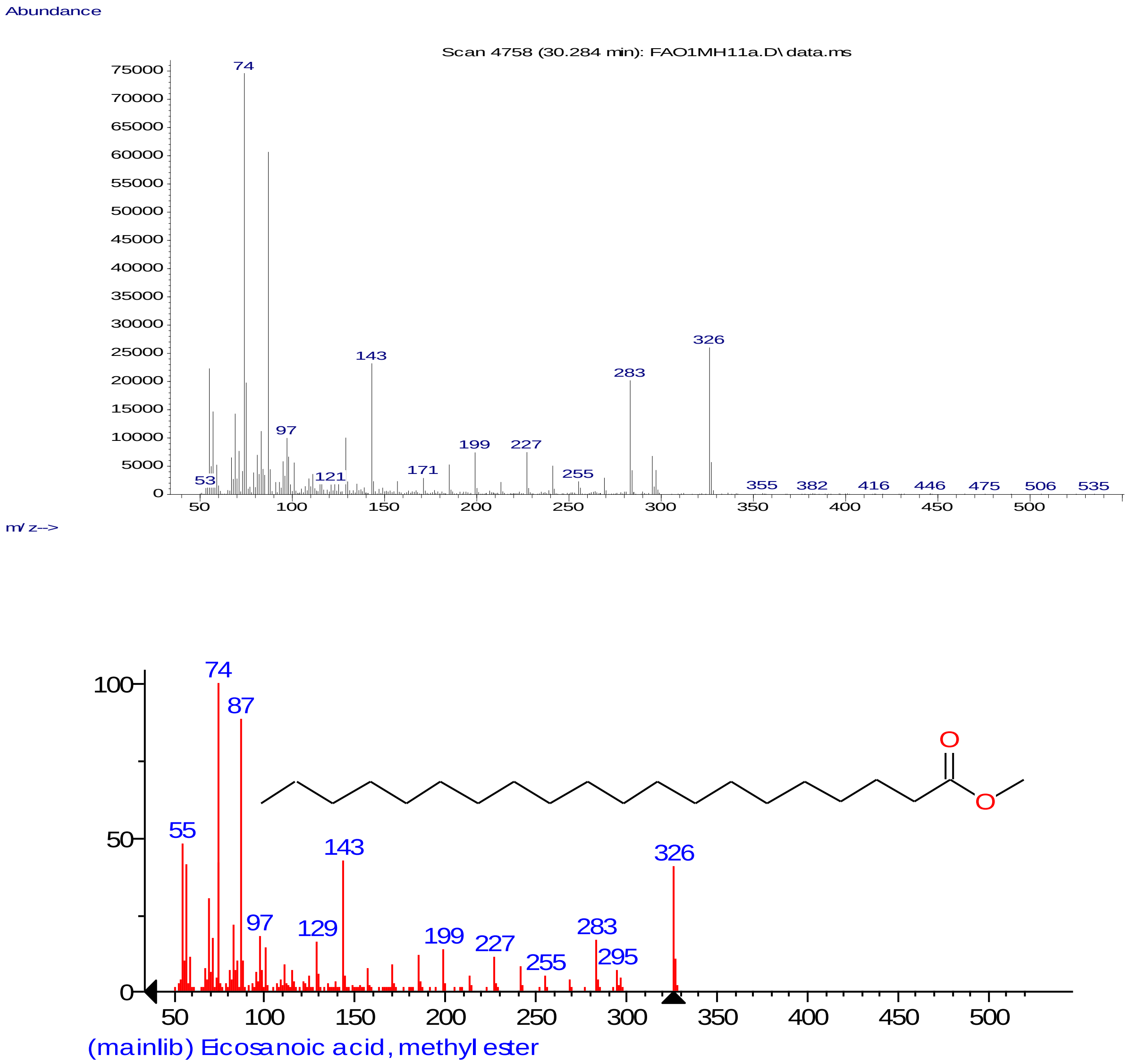

Figure P. MS spectra of arachidic acid (methyl ester) from the GC-MS analysis of the derivatized beans lipid fractions (experimental – top and from the NIST database – bottom)

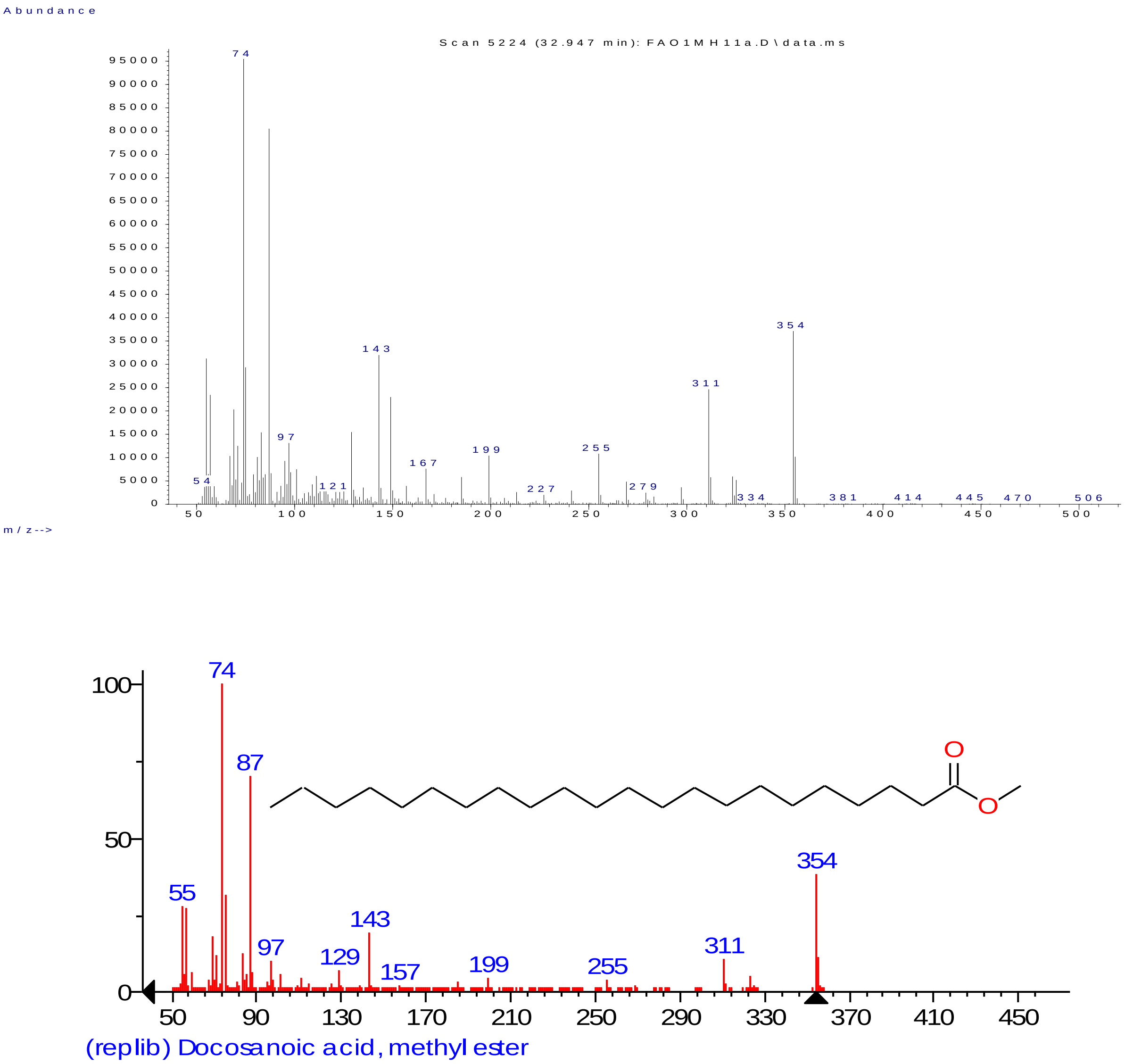

Figure Q. MS spectra of behenic acid (methyl ester) from the GC-MS analysis of the derivatized beans lipid fractions (experimental – top and from the NIST database – bottom)

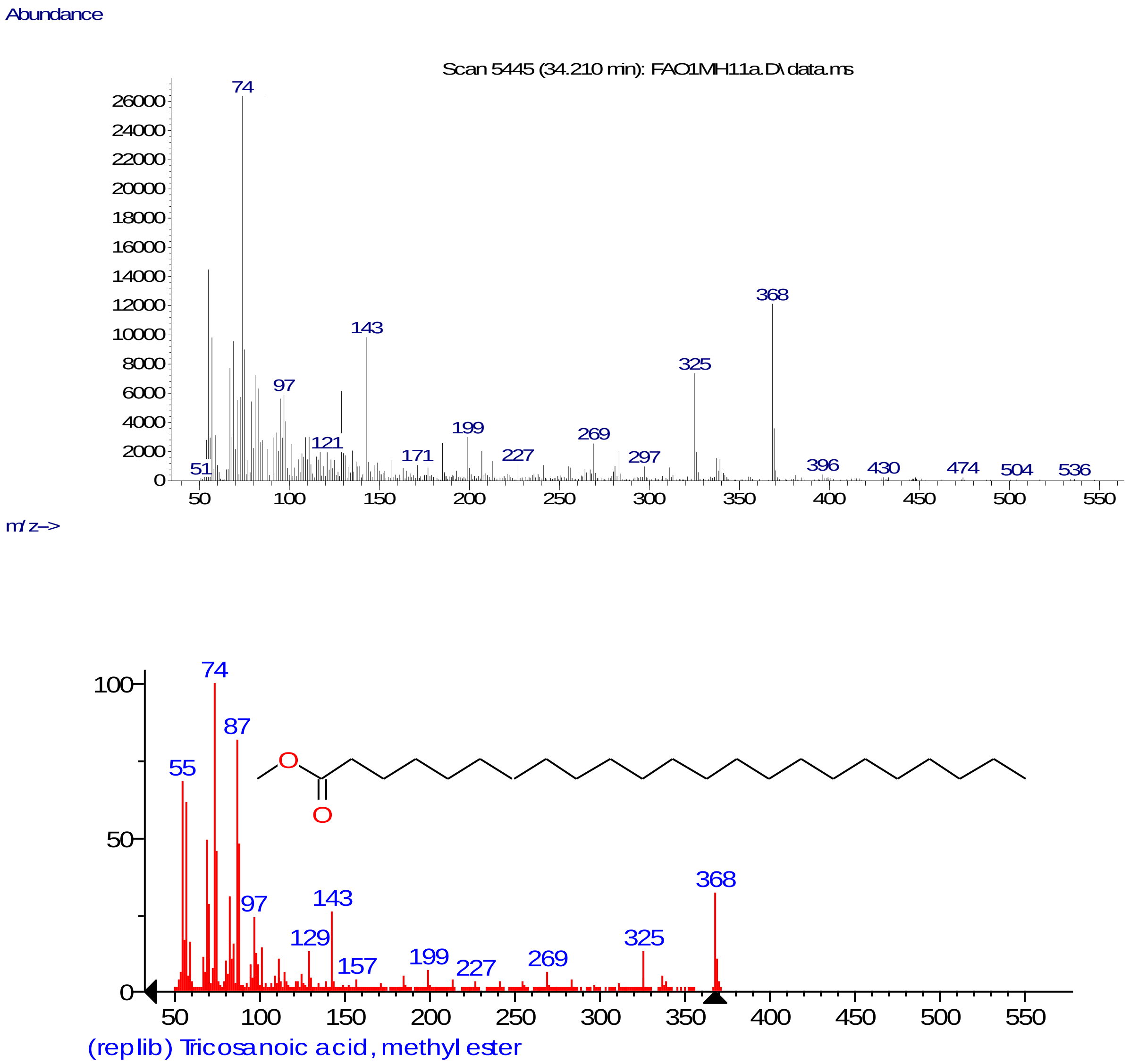

Figure R. MS spectra of tricosanoic acid (methyl ester) from the GC-MS analysis of the derivatized beans lipid fractions (experimental – top and from the NIST database – bottom)

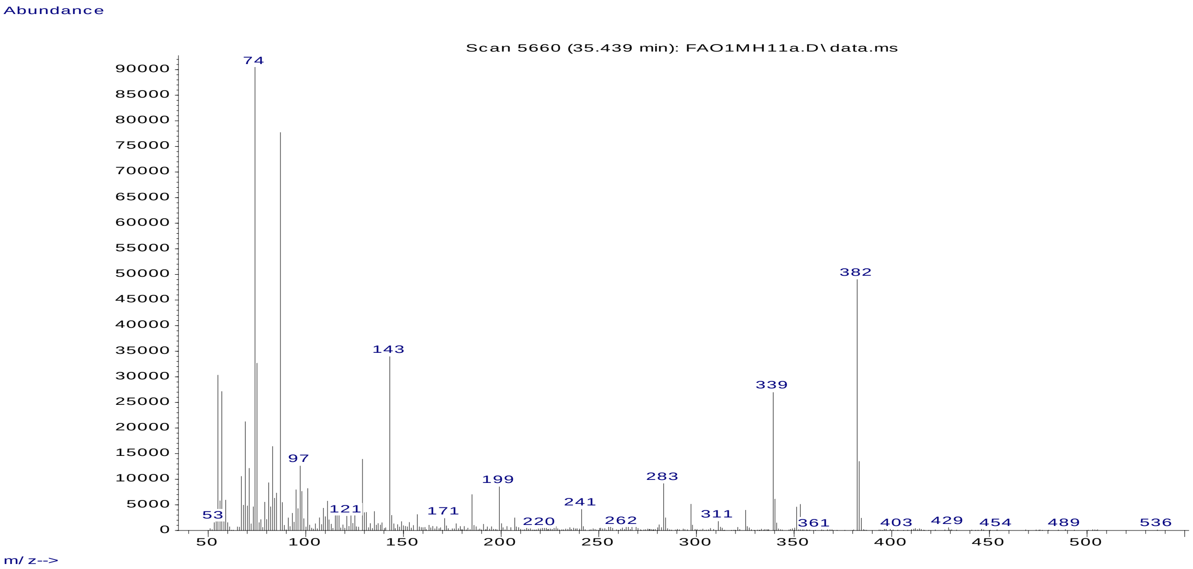

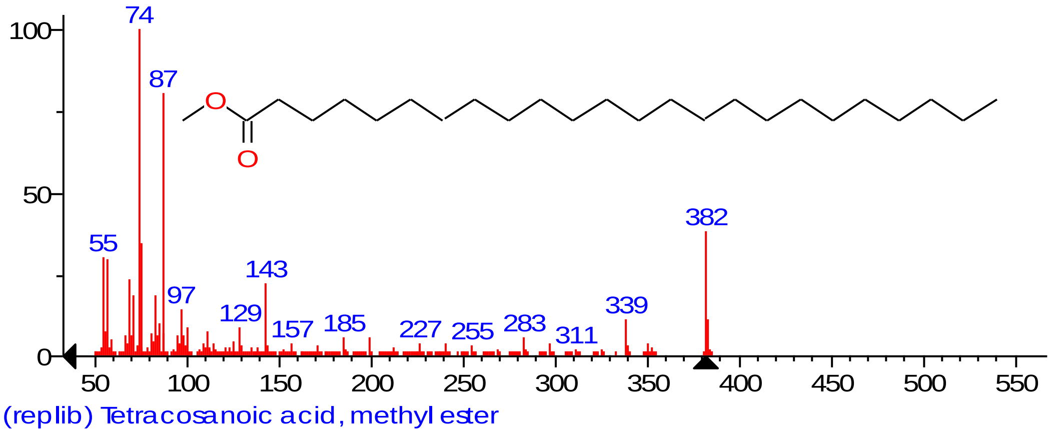

Figure S. MS spectra of lignoceric acid (methyl ester) from the GC-MS analysis of the derivatized beans lipid fractions (experimental – top and from the NIST database – bottom)

Fourier transform infrared spectroscopy (FTIR) analysis of β-cyclodextrin/common beans oil complexes

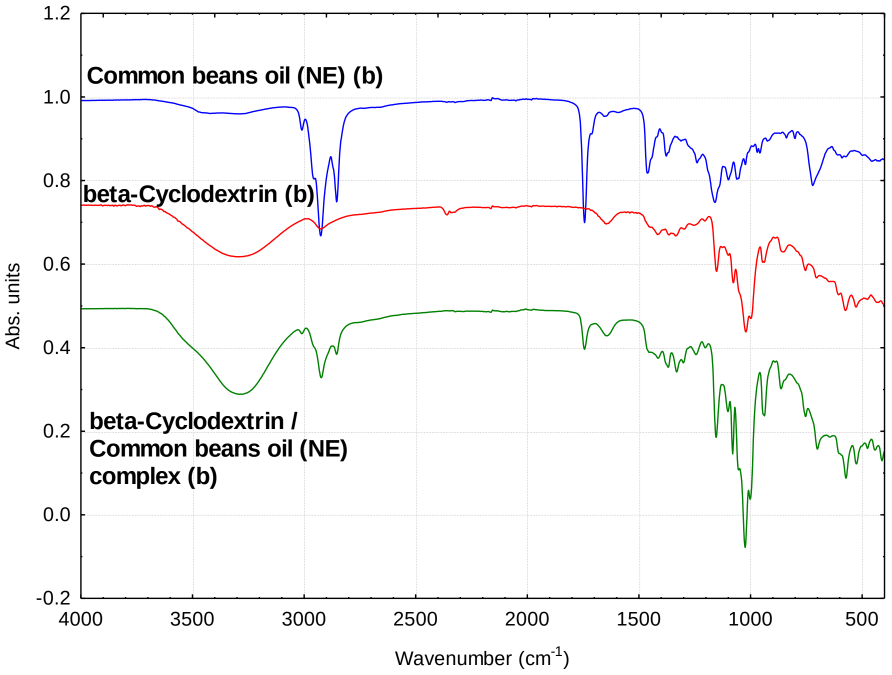

Figure T. Fourier transform infrared spectroscopy (FTIR) data for CBO-NE (blue), β-CD (red) and β-CD/CBO-NE complex (green) (duplicates "b")

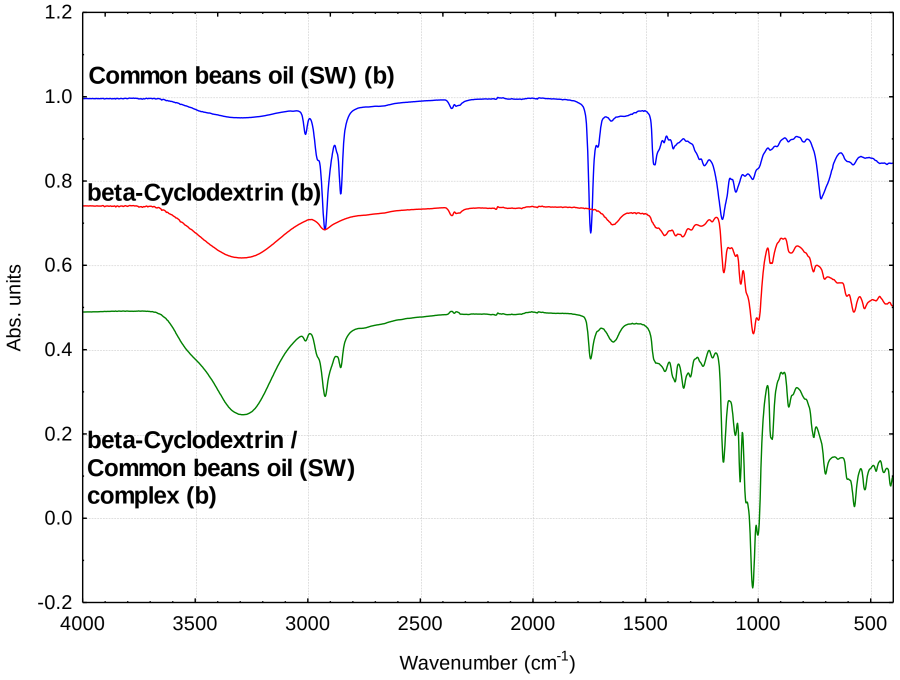

Figure U. Fourier transform infrared spectroscopy (FTIR) data for CBO-SW (blue), β-CD (red) and β-CD/CBO-SW complex (green) (duplicates "b")

Powder X-Ray diffractometry (PXRD) analysis of β-cyclodextrin/common beans oil complexes

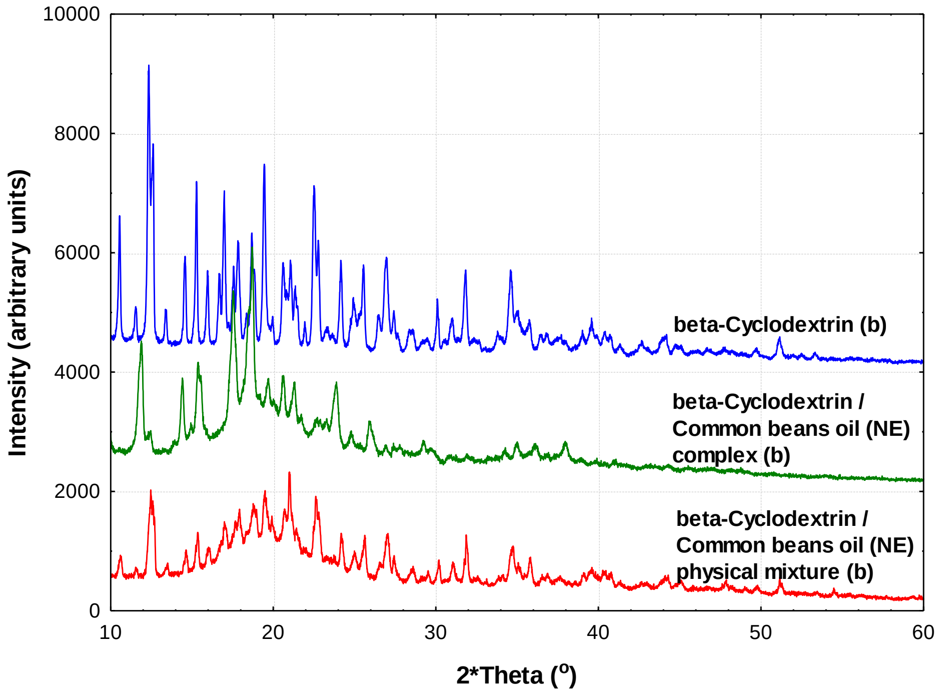

Figure V. Powder X-Ray diffractometry (PXRD) data for β-CD (blue), β-CD/CBO-NE complex (green), β-CD + CBO-NE physical mixture (red) (duplicates "b")

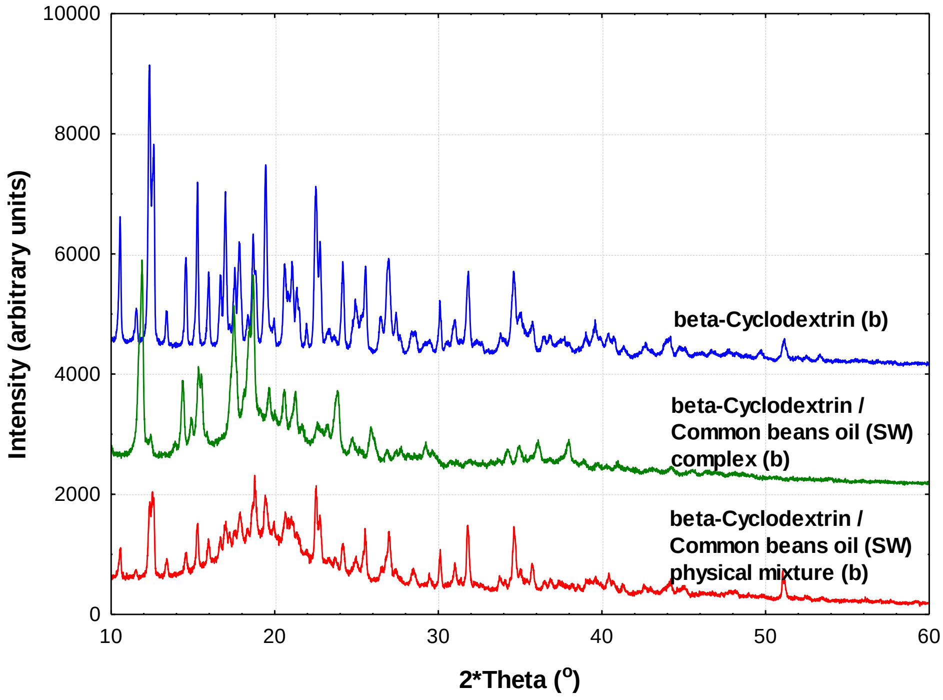

Figure W. Powder X-Ray diffractometry (PXRD) data for β-CD (blue), β-CD/CBO-SW complex (green), β-CD + CBO-SW physical mixture (red) (duplicates "b")

Thermogravimetry-Differential Thermogravimetry (TG-DTG) data of β-cyclodextrin/common beans oil complexes (duplicates "b")

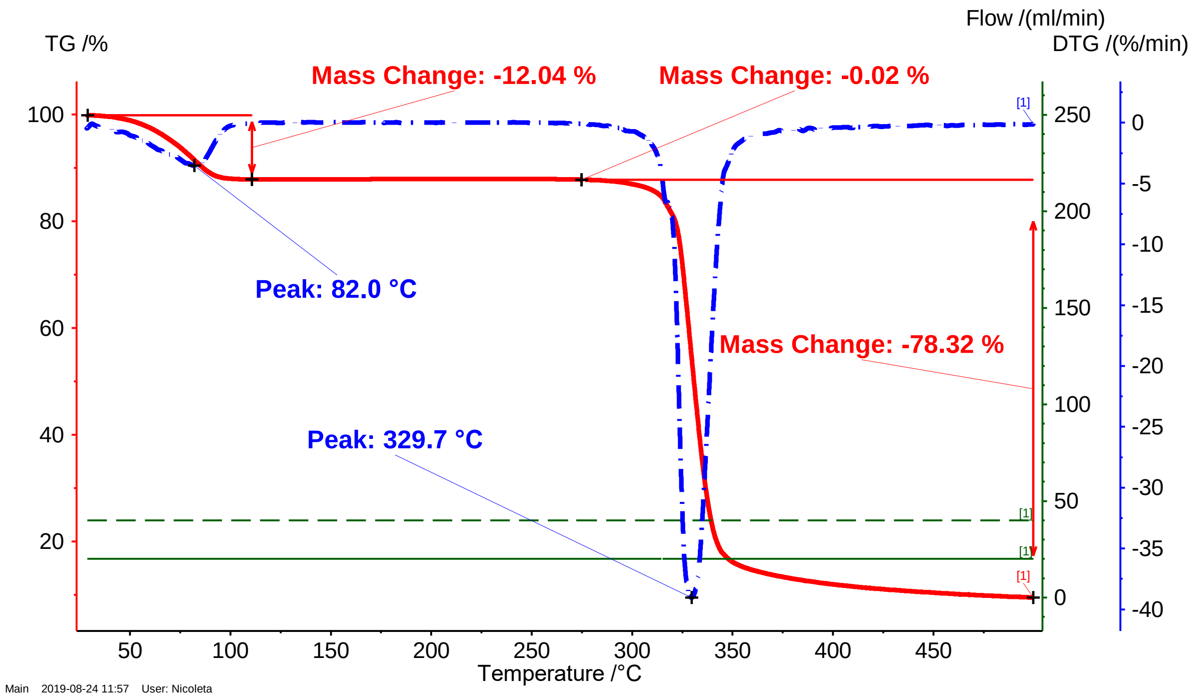

Figure X. Thermogravimetry-differential thermogravimetry (TG-DTG) analysis of β-cyclodextrin (β-CD, duplicate "b")

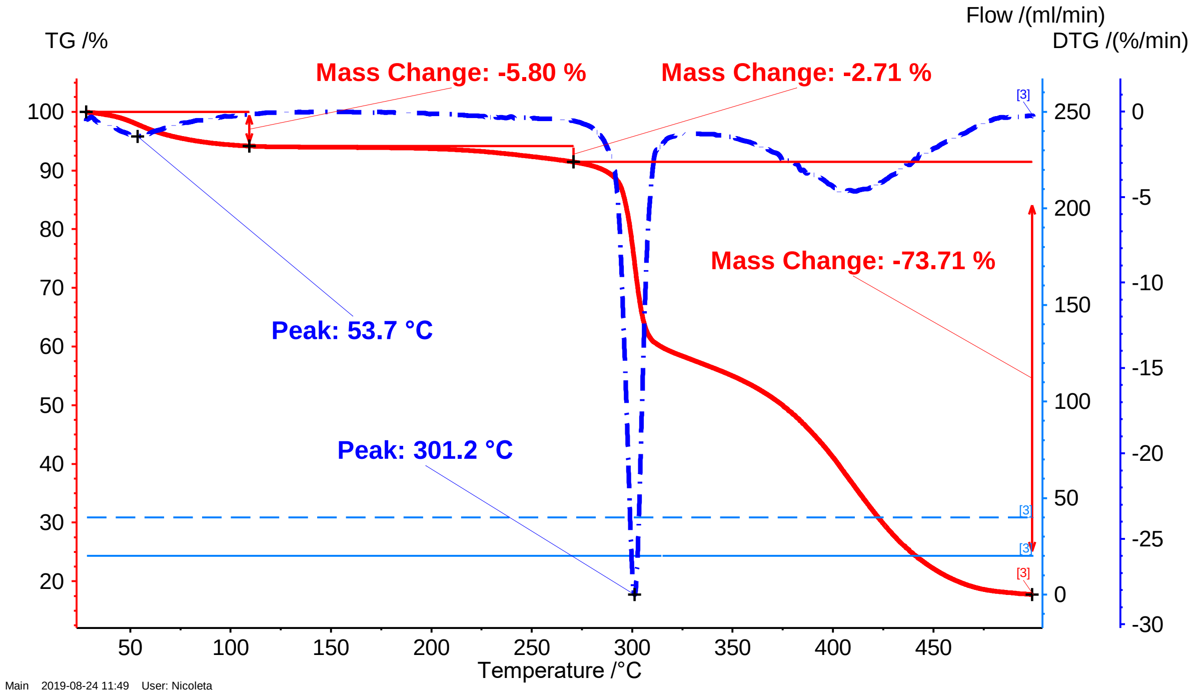

Figure Y. Thermogravimetry-differential thermogravimetry (TG-DTG) analysis of β-cyclodextrin / common beans (North-East) oil complex (β-CD / CBO-NE, duplicate "b")

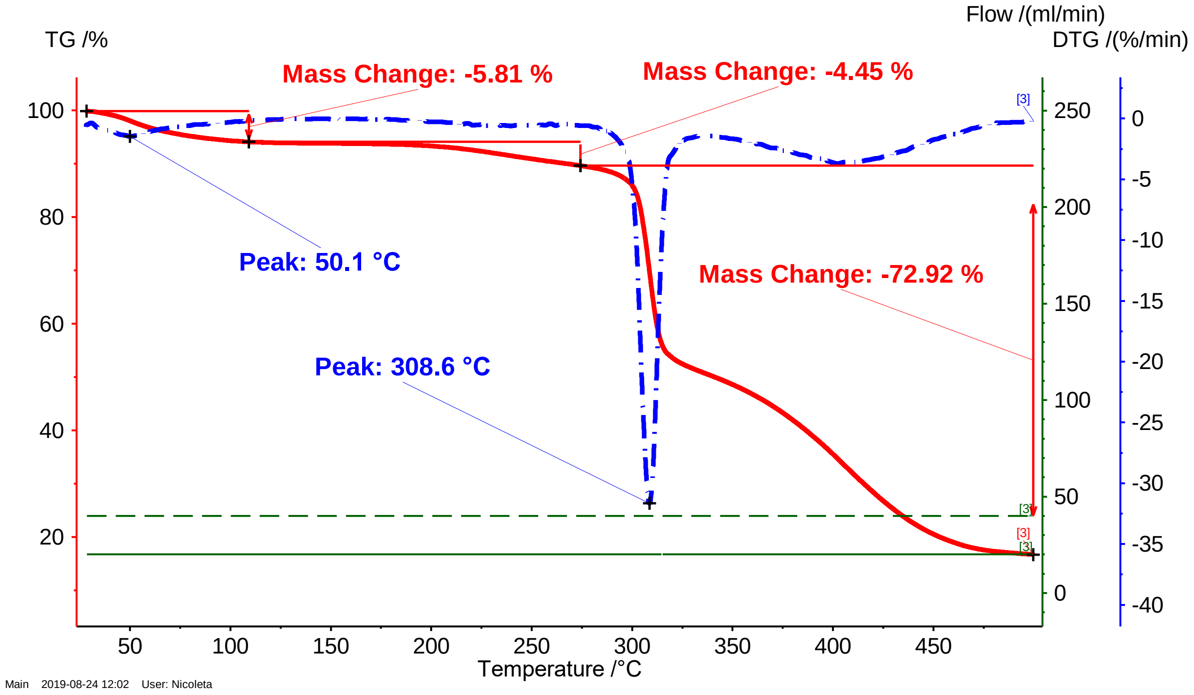

Figure Z. Thermogravimetry-differential thermogravimetry (TG-DTG) analysis of β-cyclodextrin / common beans (South-West) oil complex (β-CD / CBO-SW, duplicate "b")

Differential Scanning Calorimetry (DSC) data of β-cyclodextrin/common beans oil complexes (duplicates "b")

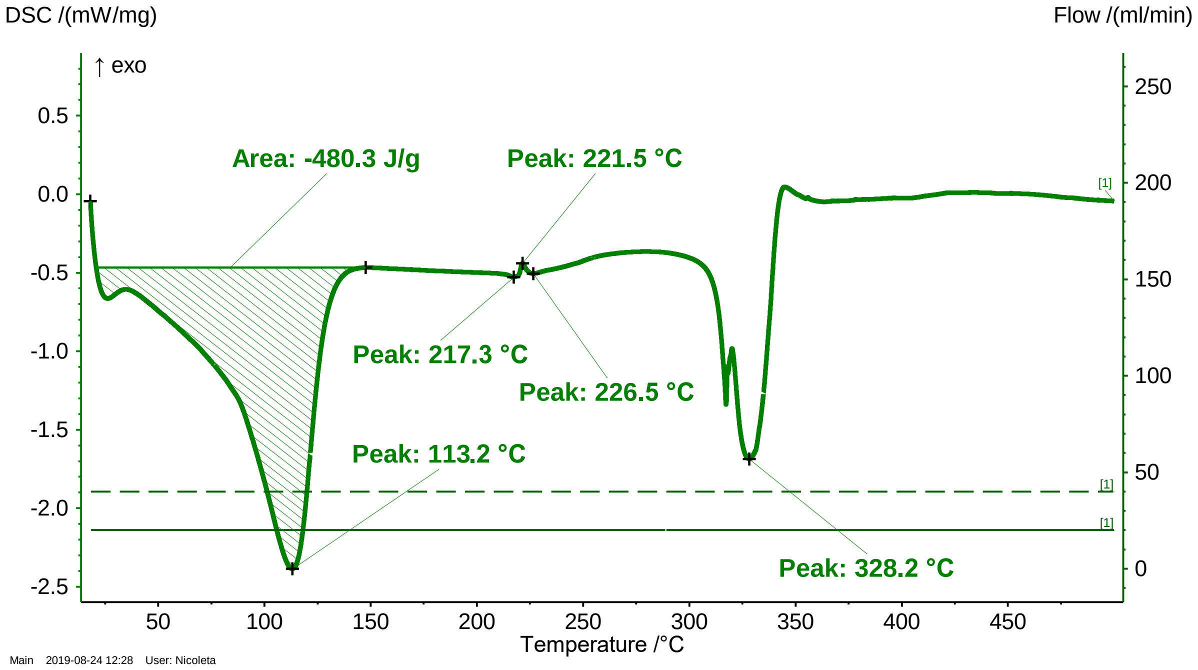

Figure AA. Differential scanning calorimetry (DSC) analysis of β-cyclodextrin (β-CD, duplicate "b")

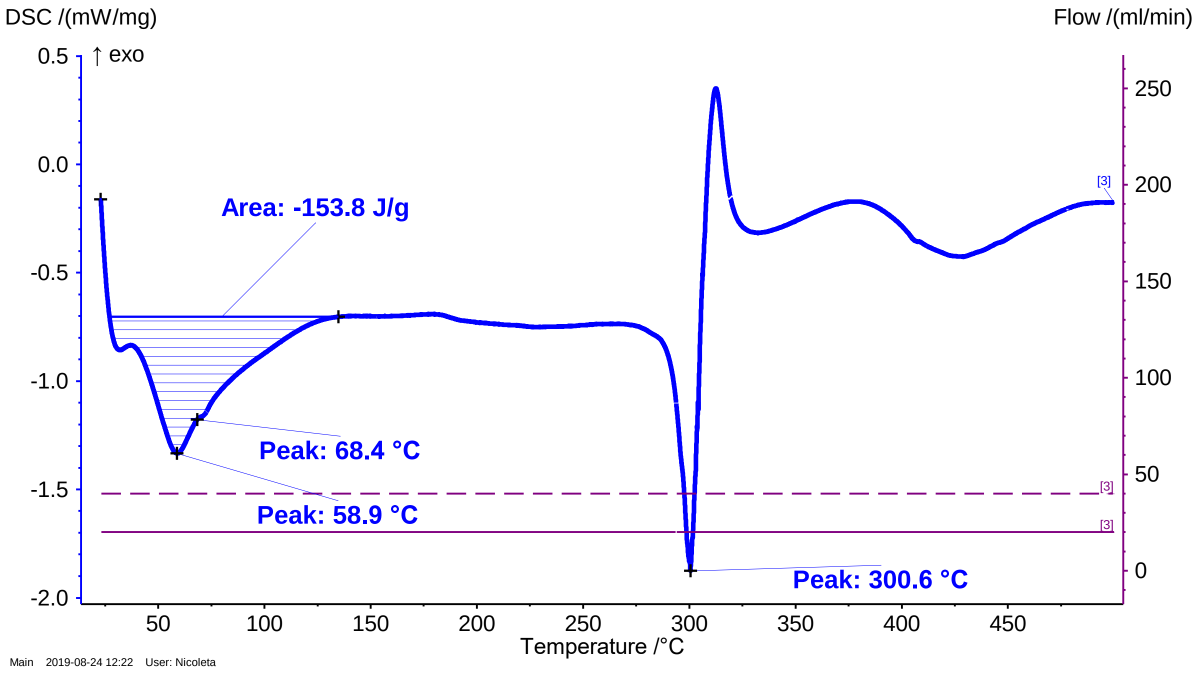

Figure AB. Differential scanning calorimetry (DSC) analysis of β-cyclodextrin / common beans (North-East) oil complex (β-CD / CBO-NE, duplicate "b")

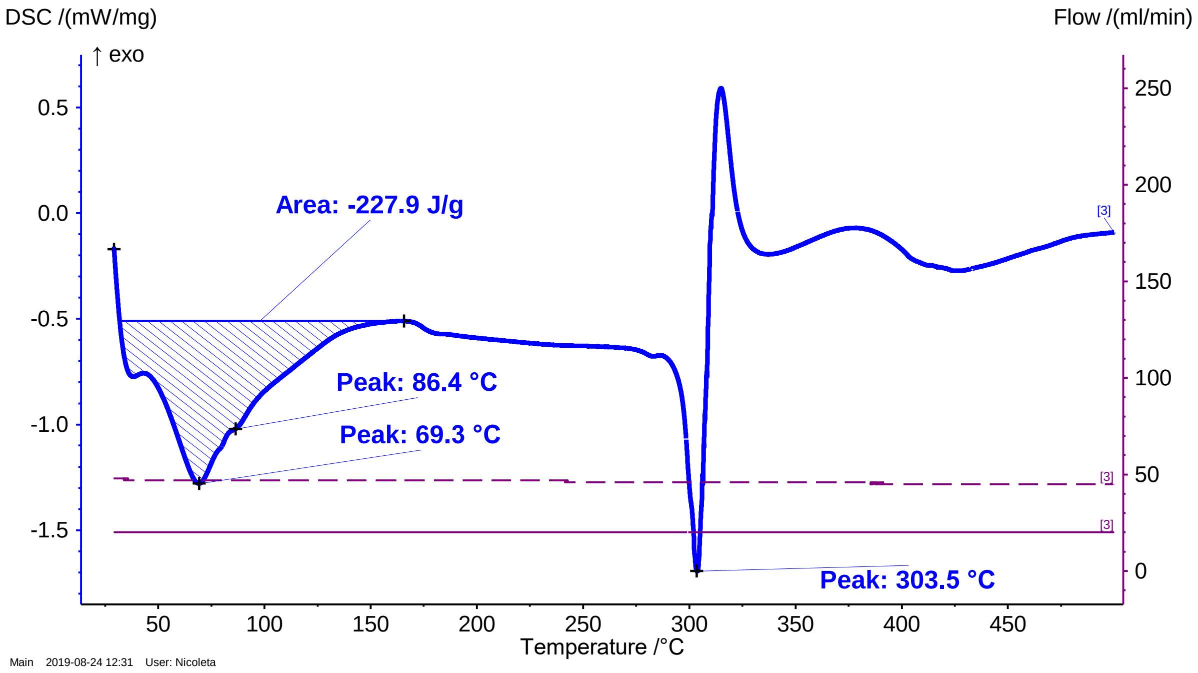

Figure AC. Differential scanning calorimetry (DSC) analysis of β-cyclodextrin / common beans (South-West) oil complex (β-CD / CBO-SW, duplicate "b")
